# Evaluating methods of inferring gene regulatory networks highlights their lack of performance for single cell gene expression data

**DOI:** 10.1186/s12859-018-2217-z

**Published:** 2018-06-19

**Authors:** Shuonan Chen, Jessica C. Mar

**Affiliations:** 1 Department of Systems and Computational Biology, Albert Einstein College of Medicine, Bronx, New York, USA; 2 Department of Epidemiology and Population Health, Albert Einstein College of Medicine, Bronx, New York, USA; 3 Australian Institute for Bioengineering and Nanotechnology, University of Queensland, Brisbane, QLD Australia

**Keywords:** Gene regulatory network, Single cell genomics, Bayesian network, Correlation network

## Abstract

**Background:**

A fundamental fact in biology states that genes do not operate in isolation, and yet, methods that infer regulatory networks for single cell gene expression data have been slow to emerge. With single cell sequencing methods now becoming accessible, general network inference algorithms that were initially developed for data collected from bulk samples may not be suitable for single cells. Meanwhile, although methods that are specific for single cell data are now emerging, whether they have improved performance over general methods is unknown. In this study, we evaluate the applicability of five general methods and three single cell methods for inferring gene regulatory networks from both experimental single cell gene expression data and in silico simulated data.

**Results:**

Standard evaluation metrics using ROC curves and Precision-Recall curves against reference sets sourced from the literature demonstrated that most of the methods performed poorly when they were applied to either experimental single cell data, or simulated single cell data, which demonstrates their lack of performance for this task. Using default settings, network methods were applied to the same datasets. Comparisons of the learned networks highlighted the uniqueness of some predicted edges for each method. The fact that different methods infer networks that vary substantially reflects the underlying mathematical rationale and assumptions that distinguish network methods from each other.

**Conclusions:**

This study provides a comprehensive evaluation of network modeling algorithms applied to experimental single cell gene expression data and in silico simulated datasets where the network structure is known. Comparisons demonstrate that most of these assessed network methods are not able to predict network structures from single cell expression data accurately, even if they are specifically developed for single cell methods. Also, single cell methods, which usually depend on more elaborative algorithms, in general have less similarity to each other in the sets of edges detected. The results from this study emphasize the importance for developing more accurate optimized network modeling methods that are compatible for single cell data. Newly-developed single cell methods may uniquely capture particular features of potential gene-gene relationships, and caution should be taken when we interpret these results.

**Electronic supplementary material:**

The online version of this article (10.1186/s12859-018-2217-z) contains supplementary material, which is available to authorized users.

## Background

Every cell in an organism is regulated by its own unique transcriptome. Advances in single cell sequencing technologies have illuminated how the regulatory processes that control individual cells consist of signals that are variable and heterogeneous. Quantifying single cell transcriptomes in large numbers has therefore allowed us to survey the landscape of heterogeneity in gene expression, resulting in the discovery of new cell sub-populations that are important for driving cellular differentiation and disease processes. It is remarkable to consider that these discoveries would otherwise be undetectable using standard approaches from bulk samples. As single cell biology continues to gain greater prominence, it is inevitable that our understanding of how signal transduction pathways operate will be updated, and that key regulators and new cell types can be identified with increased resolution [1].

The analysis of gene expression data from single cells comes with a variety of computational challenges. There are features that are inherent to single cell gene expression data, that distinguish this data type from their bulk sample counterparts, and require additional attention as far as statistical analysis and bioinformatics modeling are concerned. For this reason, computational methods that were originally developed for bulk sample data may not necessarily be suitable for data generated from single cells. For instance, single cell data has higher rates of zero values than bulk sample data. This results from a combination of true biological effects where a transcript of a gene is not expected to be produced in every cell, and technical variation, where higher degrees of sensitivity and variation are associated with single cell assays because of the limited amounts of biological material. For standard bulk sample data, it is often common to exclude or impute these zero values as a preprocessing step to improve the stability of downstream analyses. However, in a single cell setting, the higher rates of zero values mean that filtering or imputation approaches may distort the overall shape of the gene expression distribution substantially, and therefore a more careful set of preprocessing rules is required [2, 3].

Another feature of single cell data is the range of gene expression distributions that are present in a cell population. Because of heterogeneity in gene expression of single cells, these distributions may not always follow a Gaussian distribution or even a single distribution type, which is a common assumption at the core of many standard bioinformatics approaches. Analyzing single cell data therefore requires methodologies that can address these kinds of data-specific challenges to produce reliable inferences.

Recently, new methods have been developed that deal with specific aspects of analyzing single cell gene expression data. MAST [4] assesses differential gene expression while accounting for technical variation in single cell data, whereas scDD tests for differences between gene expression distributions [5]. Multiple studies show these single cell-specific methods outperform standard bulk sample methods for detecting differentially expressed genes [6, 7]. A host of other methods has been released to analyze gene expression data from single cells that go beyond differential expression [8–15]. One approach from the Monocle toolkit [16] infers the trajectory of individual cells to recreate “pseudo-time”, a mapping that provides insight into the transcriptional dynamics or developmental hierarchies of single cells, including the gene sets or cell sub-populations underlying these relationships [17–20]. These newly-developed methods show promise in their potential to improve the accuracy of inferences derived from single cell gene expression data.

In contrast to differential expression analysis, it is only recently that the methods for gene regulatory network (GRN) modeling have been developed specifically for single cell data [21]. While each method addresses some of the distinct features of single cell data, a common theme is that network reconstruction is limited to a simple model. This is a concern because the inferred networks may fail to fully represent and exploit the complexity occurring in the transcriptomes of single cells. For instance, some methods such as the single-cell network synthesis (SCNS) toolkit, as well as BoolTraineR (BTR) [22] rely on a binary indicator variable for gene expression which may be an over-simplification of more subtle expression changes and hidden interactions. Also, the computational cost of calculating a Boolean function and cell state constrains the scalability of the methods to more meaningful and realistic numbers of genes to study. More recently, a method based on a Gamma-Normal mixture model [23] shows potential for capturing the multi-modality of gene expression in single cells; however, limitations of this method are that it is only appropriate for profiles with two to three components, and must follow these two distribution types of a Gamma and Normal distribution. The network reconstruction part of this method is also based on co-activation where interactions are identified using binary activation/de-activation relationships which may not be sensitive enough to generalize across all genes. Another recent method SCODE requires pseudo-time estimates for single cell datasets to solve linear ordinary differential equations (ODEs) [24]. This may be problematic if the pseudo-time inference step introduces an additional level of noise or error that then affects the accuracy of downstream network reconstruction.

Notably, many network analyses of single cell data still depend on methods that were developed for bulk sample data, especially the popular use of co-expression networks [25–27]. These association networks are straightforward to interpret, but may not necessarily be suitable for single cell gene expression data since they do not account for drop-out events or model heterogeneity in the data. Therefore, understanding how standard network methods perform when applied to single cell data, as well as exploring whether the methods designed for single cell data have higher accuracy, are critical questions for conducting appropriate analyses. To our knowledge, a thorough investigation into the utility of these general and new network approaches for single cells has not been done. Understanding the limitations and strengths of these existing methods is informative for providing guidance for choosing a network method for single cell analysis, and the development of new network inference methods for single cell gene expression data.

**Fig. 1:**
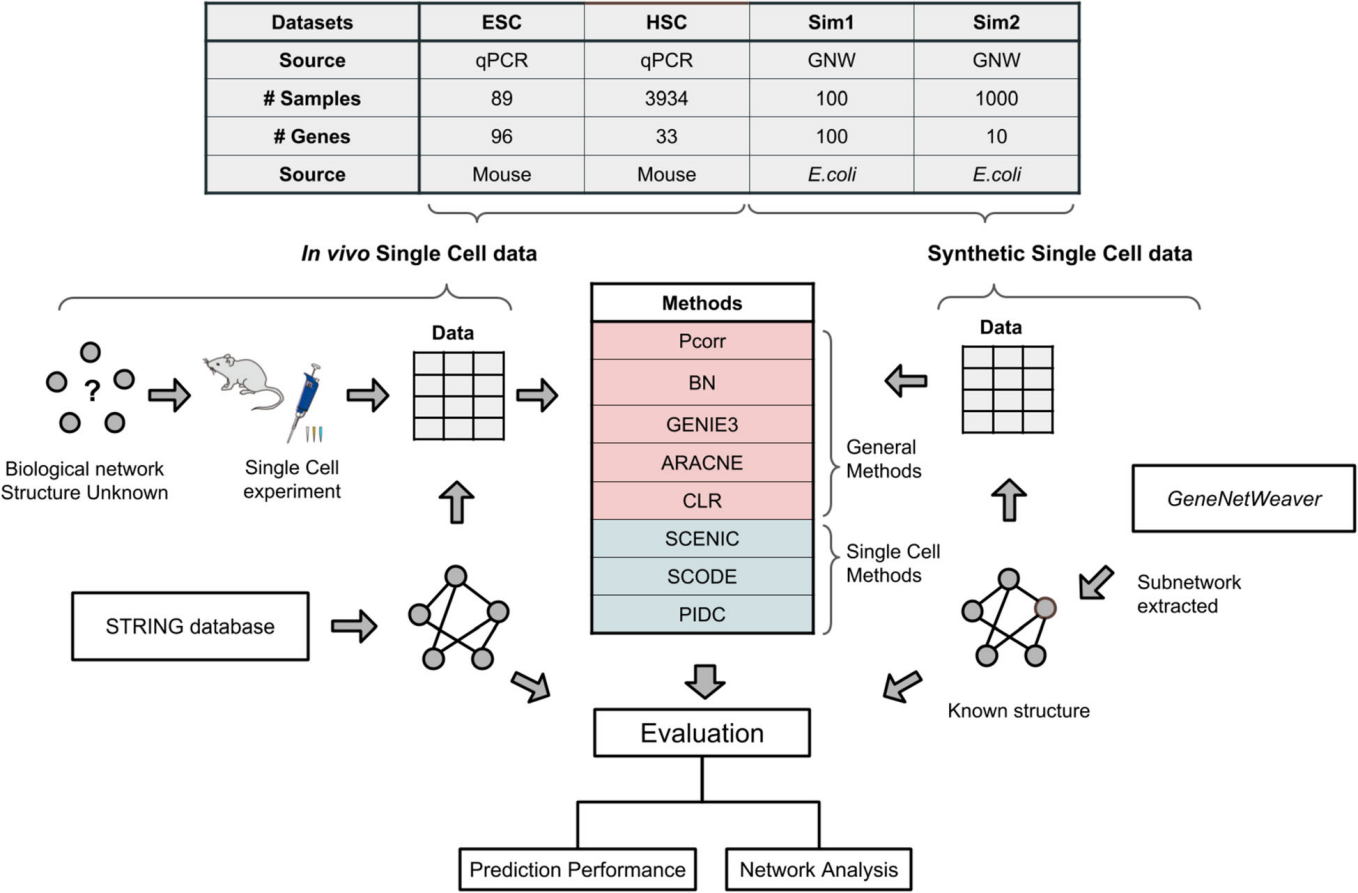
Study Workflow. Eight network reconstruction methods – including five general methods: partial correlation (Pcorr), Bayesian network (BN), GENIE3, ARACNE and CLR, and three single cell-specific methods: SCENIC, SCODE and PIDC – were applied to two single cell experimental datasets, and two simulated datasets that resemble single cell data. Evaluation of these methods was based on their ability to reconstruct a reference network, and this was assessed using PR, ROC curves, and other network analysis metrics

In this study, we investigated the performance of five commonly-used network methods originally developed for bulk sample data, plus three single cell-specific network methods, for reconstructing gene regulatory networks from single cell gene expression data (see Fig. 1 for study design). To evaluate their performance, we used both publicly-available experimental data as well as in silico simulated data where the underlying network structure is known. We show that these network methods all performed poorly for single cell gene expression data, while one of the single cell network methods performed well for simulated data. The rankings were not consistent overall amongst the four datasets. Even the one single cell method which was the best performer for the simulation datasets, did not show good performance when applied to real single cell experiment data. We also show that the networks learned from each method have characteristic differences in network topology and the predicted sets of inferred relationships. Given that very low degree of overlap was observed between different single cell methods, we suggest that single cell-specific methods have their own edge detection criterion, and therefore additional caution should be taken when choosing one network method over another, or when interpreting the results from a reconstructed network.

## Methods

### GRN inference methods

We use *N* to denote the total number of genes, and use *S* to denote the total number of samples i.e. single cells profiled. A gene expression dataset is represented by a *S* × *N* matrix, where each row vector *s* (*s* = 1, …, *S*) represents a *N*-dimensional transcriptome, and each column vector *y* (*y* = 1, …, *N*) corresponds to a *S*-dimensional gene profile in the total cell population. The goal of the network inference method is to use the data matrix (experimental or synthetic datasets) to predict a set of regulatory interactions between any two genes from the total of *N* genes. The final output is in the form of a graph with *N* nodes and a set of edges. In a GRN, each node in the network represents a gene and an edge connecting two nodes represents an interaction between these two genes (representing either direct physical connections or indirect regulation). In the next section, we describe the set of network inference methods that were used in our study, followed by the description of datasets used, the reference networks, and statistical metrics used to assess performance.

#### Partial correlation (Pcorr)

The principle underlying correlation networks is that if two genes have highly-correlated expression patterns (i.e. they are co-expressed), then they are assumed to participate together in a regulatory interaction. It is important to highlight that co-expressed genes are indicative of an interaction but this is not a necessary and sufficient condition. Partial correlation is a measure of the relationship between two variables while controlling for the effect of other variables. For a network structure, the partial correlation of nodes ***X***_***i***_ and ***X***_***j***_ (i-th and j-th gene) are defined with respect to other nodes ${\boldsymbol{X}}_{{\boldsymbol{S}}_{\boldsymbol{m}}}$, where ***S***_***m***_ ***∈ X***_\{***i***, ***j***}_:

$$ {\rho}_{ij\mid {S}_m}={\mathrm{corr}}_{X_i{X}_j\mid {S}_m}=\frac{\sigma_{ij\mid {S}_m}}{\sqrt{\sigma_{ii\mid {S}_m}{\sigma}_{jj\mid {S}_m}}} $$

Under this definition, inferring the Pcorr is equivalent to inferring a the set of non-zero partial correlations between variables, and testing the hypothesis:

$$ {\displaystyle \begin{array}{c}{H}_0:{\rho}_{ij\mid {S}_m}=0\\ {}\mathrm{vs}.\\ {}{H}_1:{\rho}_{ij\mid {S}_m}\ne 0,\end{array}} $$

where ${\rho}_{ij\mid {S}_m}$ indicates the partial correlation coefficient defined above. Therefore the presence of an edge between *x*_*i*_ and *x*_*j*_ indicates that a correlation exists between *x*_*i*_ and *x*_*j*_regardless of which other nodes are being conditioned on.

Typically, gene expression profiles from single cell data follow an analog-like, multimodal distribution rather than a unimodal continuous shape. Therefore, metrics like the Pearson correlation coefficient are less suited for single cell expression data because this metric measures a linear dependency between two variables. Therefore, a more appropriate measure is a rank-based measure of correlation, such as the Spearman correlation and Kendall rank correlation coefficients. Given the non-linear nature of single cell gene expression data, Spearman’s correlation coefficient was used in this study. Pairwise partial correlations were calculated, and Fisher’s transformation was used for variance stabilization:

$$ {z}_{ij\mid {S}_m=}\frac{1}{2}\log \frac{1+{\rho}_{ij\mid {S}_m}}{1-{\rho}_{ij\mid {S}_m}} $$

Adjustment for multiple testing correction of the *P*-values was done using the Benjamini-Hochberg method [28]. Statistical significance was defined at the 0.05 level, and this threshold was used to identify the final set of predicted pairwise interactions using Pcorr.

#### Bayesian networks

A Bayesian network (BN) encodes conditional dependencies between random variables *X*, that are represented as nodes in the graph or network. Each node is characterized by a conditional probability table for discrete data, or a regression model in the case of continuous variables, that specify the probability or likelihood of obtaining a certain outcome for the node given the values of its parent nodes.

The network structure is defined as the graph *G* = (*V*,*E*), where *V* corresponds to the set of random variables *X*, represented as nodes, and *E* corresponds to the set of edges that connect any of these nodes in the graph. In this study, we only consider a BN for continuous variables since gene expression is more appropriately modeled as a continuous measure. Under this setting, BN defines a factorization of the joint probability distribution of *V* = {*x*_1_, …*x*_*N*_} (global probability distribution) into a set of local probability distributions, given by the Markov Property of BNs, which states that each variable node directly depends on its parent variables ${\varPi}_{X_i}$:

$$ f\left({x}_1,\dots, {x}_N\right)=\prod \limits_{i=1}^Nf\left({x}_i|{\varPi}_{x_i}\right) $$

Because gene expression is typically modeled as a continuous value, *Gaussian Bayesian Networks* are commonly used to reconstruct networks for this kind of data. In such a BN, the global distribution is assumed to follow a multivariable Normal distribution, and local distributions are linear regression models where the parent nodes are used as explanatory variables.

Structure learning in BN pertains to the task of learning the network structure from the dataset. There are several methods available for the task, and we used a score-based structure learning algorithm, specifically the Bayesian Information criterion (BIC) score to guide the network inference process. We used bootstrap resampling to learn a set of *R* = 1000 network structures, and then used model averaging to build an optimal single network (the significant threshold was determined by the function *averaged.network* from the R package *bnlearn* [29], which finds the optimal threshold based on the likelihood of the learned network structure). Although a BN can learn directed edges, all directions were not included in our results to facilitate a fairer comparison with the other network methods, since most of these do not infer directed edges. For this comparison, we therefore treated the directed edges showing higher absolute values as the representative regulatory relationships. BN inference was performed using the R package *bnlearn* [29].

#### GENIE3

GEne Network Inference with Ensemble of Trees (GENIE3) uses a tree-based method to reconstruct GRNs, and has been successfully applied to high-dimensional datasets [30]. It was also the best performer in the DREAM4 In Silico Multifactorial challenge [31]. In this method, reconstructing a GRN for *N* genes is solved by decomposing the task into *N* regression problems, where the aim is to determine the subset of genes whose expression profiles are the most predictive of a target gene’s expression profile.

Each tree is built on a bootstrapped sample from the learning matrix, and at each test node, *k* attributes are selected at random from all candidate attributes before determining the best split. By default, and as suggested from the original literature, $\boldsymbol{k}=\sqrt{\boldsymbol{N}}$ was used in this study. For each sample, the learning samples are recursively split with binary tests based each on a single input gene. The learning problem is equivalent to fitting a regression model, where the subset of genes are covariates, that minimizes the squared error loss between the predicted and observed expression value for the target gene. Each model produces a ranking of the genes as potential regulators of a target gene. Ranks are assigned based on weights that are computed as the sum of the total variance reduction of the output variable due to the split, and therefore indicate the importance of that interaction for its prediction of the target gene’s expression. Although GENIE3 is able to learn the directions of edges too, we used the same rationale and procedure as for the BN, where directed edges were not incorporated into the learned networks to facilitate a more straightforward comparison of results from all network methods.

#### ARACNE

Algorithm for the Reconstruction of Accurate Cellular Networks (ARACNE) [32] is one of the most common information-theoretic network approaches that is based on Mutual Information (MI). MI is a generalization of the pairwise correlation coefficient, and measures the degree of dependency between two variables ***x***_***i***_**and** ***x***_***j***_**:**

$$ \boldsymbol{M}{\boldsymbol{I}}_{\boldsymbol{i}\boldsymbol{j}}=\sum \limits_{{\boldsymbol{x}}_{\boldsymbol{i}}}\sum \limits_{{\boldsymbol{x}}_{\boldsymbol{j}}}\boldsymbol{p}\left({\boldsymbol{x}}_{\boldsymbol{i}},{\boldsymbol{x}}_{\boldsymbol{j}}\right){\mathbf{\log}}_{\mathbf{2}}\frac{\boldsymbol{p}\left({\boldsymbol{x}}_{\boldsymbol{i}},{\boldsymbol{x}}_{\boldsymbol{j}}\right)}{\boldsymbol{p}\left({\boldsymbol{x}}_{\boldsymbol{i}}\right)\boldsymbol{p}\left({\boldsymbol{x}}_{\boldsymbol{j}}\right)}, $$

where *p*(*x*_*i*_, *x*_*j*_) is the joint probability distribution of *x*_*i*_ and *x*_*j*_, and *p*(*x*_*i*_) and *p*(*x*_*j*_) are the marginal probability distribution functions of *x*_*i*_ and *x*_*j*_, respectively. To calculate MI, discrete variables are required. We used the R package *minet*, which calculates MI by equal-width binning for discretization and empirical entropy estimation as described in [33]. Following the calculation of MI for every available pair of genes, ARACNE applies the Data Processing Inequality (DPI) to eliminate indirect effects that can be explained by the remaining interactions in the network. DPI states that if gene *x*_*i*_ interacts with gene *x*_*k*_ via gene *x*_*j*_, or equivalently:

$$ {x}_i\to {x}_j\to {x}_k, $$

then,

$$ I\left({x}_i,{x}_k\right)\le \min\ \left\{I\left({x}_i,{x}_j\right),I\left({x}_j,{x}_k\right)\right\}. $$

ARACNE calculates all pairwise MIs, and for all possible gene triplets, it will remove those interactions that violate the DPI beyond a specified level of tolerance given by *eps*, which is a parameter designed to compensate for errors in the estimated MI. Specifically, if the difference between the potential indirect interaction and the minimum of any other two is such that:

$$ I\left({x}_i,{x}_k\right)-\min\ \left\{I\left({x}_i,{x}_j\right),I\left({x}_j,{x}_k\right)\right\}> eps. $$

then the potential edge (connecting *x*_*i*_ and *x*_*k*_) will be labeled as an indirect interaction and be removed from the inferred network. The tolerance threshold *eps* was set to *eps* = 0.1 for all network inference with ARACNE (a value of *eps* = 0.1–0.2 is suggested in the original paper).

#### CLR

Context Likelihood of Relatedness (CLR) [31, 34] is another commonly-used approach that is also based on MI. The difference is that CLR takes into account the background distribution of the MI values where ARACNE does not. The adjustment for the background distribution is aimed to reduce the prediction of false positives in the detection of interactions that may be caused by noise. Similar for ARACNE, we used the R package *minet* for the entropy and MI calculation. CLR derives a modified *z*-score that is associated with the empirical distributions of the MI for each ***i***:

$$ {\boldsymbol{z}}_{\boldsymbol{i}}=\underset{\boldsymbol{j}}{\mathbf{\max}}\left\{\mathbf{0},\frac{\boldsymbol{I}\left({\boldsymbol{x}}_{\boldsymbol{i}},{\boldsymbol{x}}_{\boldsymbol{j}}\right)-{\boldsymbol{\mu}}_{\boldsymbol{i}}}{{\boldsymbol{\sigma}}_{\boldsymbol{i}}}\right\} $$

where *μ*_*i*_ and *σ*_*i*_ are the mean and standard deviation of MI values *I*(*x*_*i*_, *x*_*k*_), *k* = 1, …*X*. The pairwise interaction likelihood score is then estimated between two genes *x*_*i*_ and *x*_*j*_ based on the joint likelihood, which is used as the weight of the edges in constructing the final network:

$$ {\omega}_{ij}=\sqrt{z_i^2+{z}_j^2.} $$

#### SCENIC

Single-Cell rEgulatory Network Inference and Clustering (SCENIC) is a recently-released single cell method for identifying stable cell states and network activity based on the estimated GRN model [35]. This GRN reconstruction uses gene co-expression modules (which can be inferred from GENIE3, for example), combined with known cis-regulatory motif enrichment analysis. Specifically, it borrows information from a pre-built database (RcisTarget), to identify enriched transcription factor binding motifs in the identified co-expression modules. Significantly-enriched motifs are then associated with their corresponding upstream transcription factors. The genes from any enriched motifs for the same upstream transcription factors are combined. Top-ranked genes for each motif are selected as the regulon, and each transcription-regulon combination is assigned in the edge list to obtain the network.

As suggested by the method’s authors, we incorporated the transcription factor information from RcisTarget when GENIE3 was applied to reconstruct the co-expression module network. However, since the weights of each edge in SCENIC are derived from the GENIE3 algorithms, we did not incorporate ranking results for SCENIC in analyses that involved GENIE3. Instead, results from SCENIC were compared with networks inferred by other single cell methods. Also, since this method is based on RcisTarget, which only provides databases from human and mice, we only applied this method to the two single cell experimental data, as data generated from the simulation only contain *E. coli* genes.

#### SCODE

SCODE is a method developed to reconstruct a GRN for single cell data via regulatory dynamics based on ODEs [24]. Specifically, the expression dynamics of transcription factors are described using linear ODEs:

$$ d\mathrm{x}=\mathrm{Ax} dt, $$

where A corresponds to the square matrix representing the regulatory relationships between variables (i.e., weighted adjacency matrix corresponding to the reconstructed network). SCODE aims to optimize A with limited computational cost, so that the above equation can represent the molecular dynamics at a certain measurement point. In order to do this, pseudo-time data is required as an extra input, in addition to the expression data. We followed the method described in the original publication and used Monocle2 [16] for single cell pseudo-time estimation. For the input arguments of SCODE, we used D = 4 and *I* = 100, where D represents the number of expression patterns for the genes and *I* represents the number of iterations for the optimizations.

#### PIDC

Partial Information Decomposition and Context (PIDC) is a method developed for single cell gene expression data that uses multivariate information measures to identify potential regulatory relationships between genes [36]. Partial information decomposition (PID) was introduced to measure statistical dependencies in a triplet of variables simultaneously, by partitioning the information provided by two sources of variables about another target variable as three categories: redundant, unique, and synergistic [37]. The PIDC inference algorithm uses a measure of the average ratio of unique information between two variables across all of the third variables in the rest of the variables, i.e., $\frac{Uniqu{e}_z\left(X;Y\right)}{I\left(X;Y\right)}$, followed by the definition of Proportional Unique Contribution (PUC):

$$ {u}_{X,Y}={\sum}_{Z\in {S}_{\backslash \left\{X,Y\right\}}}\frac{{\mathrm{Unique}}_Z\left(X;Y\right)}{I\left(X;Y\right)}+{\sum}_{Z\in {S}_{\backslash \left\{X,Y\right\}}}\frac{{\mathrm{Unique}}_Z\left(Y;X\right)}{I\left(X;Y\right)} $$

That is, the sum of $\frac{Uniqu{e}_z\left(X;Y\right)}{I\left(X;Y\right)}$ calculated using every other gene Z in the network (*S* is the complete set of genes). The confidence of an edge, which is the sum of the cumulative distribution functions of all the scores for each gene, is next calculated as follows:

$$ c={F}_X\left({u}_{X,Y}\right)+{F}_Y\left({u}_{X,Y}\right) $$

where *F*_*X*_(*U*) is the estimated empirical probability distribution for all the PUC scores involving gene X. By incorporating the distribution of PUC score for a particular gene, rather than simply keeping edges that ranked highest across all genes, PIDC aims to detect the most important set of inferred interactions.

### Data and analytic methods

#### Experimental single cell datasets

The details of the datasets used in this study are summarized in Table 1. The two experimental single cell datasets were obtained from studies that profiled embryonic stem cell (ESC) populations and blood-forming stem cell populations (which we refer to as hematopoietic stem cell (HSC) to distinguish it from the former dataset) [26, 38]. These two datasets were generated using quantitative PCR from 96.96 array chips (ESC) and 48.48 array chips (HSC) on the Fluidigm BioMark HD platform.

**Table 1:** Summary of datasets used in the evaluation of the eight network methods

Datasets	ESC	HSC	Sim1	Sim2
#Sample (S)	89	3934	100	1000
#Gene (N)	96	33	100	10
Methods	Fluidigm qPCR	Fluidigm qPCR	GNW in silico	GNW in silico
Source	Mouse	Mouse	*E. coli*	*E. coli*
Reference (Ref)	STRING PPI	STRING PPI	GNW	GNW
#Edges in Ref	409	126	263	9
Citation	[26]	[38]	[40, 47]	[40, 47]

#### Reference networks derived from experimental assays

Protein-Protein Interaction (PPI) networks were extracted from the STRING database and used as a reference network to compare the reconstructed networks. These networks represent potential interactions that are derived from evidence based on experimental results, metabolic and signal transduction pathway databases, text-mining and other sources of information [39] . Of note, the reference used in our study was different from the stringent “gold standards” used in DREAM5 challenge, since we included all possible interactions and did not restrict the network to the direct regulatory interactions only. For instance, edges were permitted in the reference network if they represented protein-protein associations or a shared function between two proteins, and did not necessarily represent a physical binding event.

#### Simulated single cell datasets and in silico reference networks

Simulated datasets were generated using the software *GeneNetWeaver* (GNW), which has become a common tool to generate gene expression data and GRN model evaluations [40]. Generating datasets where the network is known provides a straightforward approach for scoring the reconstructed networks. GNW has previously been used to evaluate different GRN modeling methods. For instance, it was selected to generate the “gold standard” networks for DREAM4 and DREAM5 network inference challenges, as well as other publications that conducted comparisons of network modeling approaches [31, 41, 42].

To obtain a reference network, GNW was used to extract the topology of a subnetwork with a total number of 100 and 10 genes for two simulated datasets (Sim1 and Sim2, respectively) from the transcriptional regulatory networks of *Escherichia coli (E.coli)* that were derived from experimental data, and then expression datasets were generated by simulations based on stochastic differential equations. Since we wanted to generate two cases corresponding to real single cell experimental studies with *n = p* and *n > > p*, *S* = 100 for Sim1 and *S* = 1000 for Sim2 were generated as time series experiments in GNW. The single time point is considered as a single cell sample, and we generated the dataset of 10 time points 100 times (i.e., in total, there are 100 time series data, each with 10 time points) to obtain S = 1000 for Sim2 dataset (i.e., 10 genes and 1000 samples), while for Sim1 dataset, we sampled 100 time points from a single time series simulation (see Additional file 1: Figure S1 for simulation settings in GNW). Both Sim1 and Sim2 have the same duration of time series 1000. More detail on the processes used in our study can be found in [31]. These simulation parameters were designed to follow those similar to other studies that use in silico single cell gene expression data [36].

Since the aim of this study is to test the applicability of network inference methods to single cell data, we used the data simulated from GNW to mimic the characteristics of single cell experimental data. Considering that drop-out events are one of the most important features of single cell data, we artificially induced drop-out events to the data generated from GNW. Specifically, for each gene, we measured its population mean expression across cell samples, and used this value as a threshold. For each sample, if the gene’s expression was lower than the threshold, it would be replaced according to a Binomial probability of 0.5 (i.e., inducing drop-out where the resulting value was now either 0 or the original data point). This approach is similar to the method used to generate single cell simulation data for network evaluation that was published recently [36]. This simulated data does not perfectly represent the data distribution of an experimental single cell dataset (Additional file 2: Figure S2C & D); however, given the fact that more genuine single cell simulations are currently unavailable, this represents the current best option for simulation in this study, especially by accounting for drop-out events to mimic experimental single cell data.

#### Statistical metrics to evaluate network performance

To evaluate the performance of the network methods, the standard metrics, Precision-Recall (PR) curve and Receiver Operating Characteristic (ROC) curve were used**.** The True Positive Rate (TPR), False Positive Rate (FPR), precision and recall for ROC and PR curve were defined as functions of cut-off (k) as follows:

$$ {\displaystyle \begin{array}{l}\mathrm{TPR}\left(\mathrm{k}\right)=\mathrm{recall}\left(\mathrm{k}\right)=\frac{\mathrm{TP}\left(\mathrm{k}\right)}{\mathrm{TP}(k)+\mathrm{FN}\left(\mathrm{k}\right)}\\ {}\mathrm{FPR}\left(\mathrm{k}\right)=\frac{\mathrm{FP}\left(\mathrm{k}\right)}{\mathrm{FP}(k)+\mathrm{TN}\left(\mathrm{k}\right)}\\ {}\mathrm{precision}\left(\mathrm{k}\right)=\frac{\mathrm{TP}\left(\mathrm{k}\right)}{\mathrm{TP}(k)+\mathrm{FP}\left(\mathrm{k}\right)}\end{array}} $$

The Area Under Curve (AUC) of the PR curve (defined as AUPR) and ROC curve (defined as AUROC) were calculated using the R package *minet*. Each network method produced a weighted adjacency matrix (or an edge list which can be equivalently transformed into an adjacency matrix) for each network. For Pcorr, each value in the matrix was the inverse of the adjusted *P*-value for that pairwise correlation. For BN, each value in the matrix was the proportion of an edge to be detected in the 1000-bootstrap sampling. For GENIE3, each value was the weight that gives the the predictive importance of the link between two genes. For ARACNE, each value was the MI after processing DPI to remove any potential indirect interaction, and for CLR, each value was the *z*-score that was corrected by the MI background distribution. For SCODE, each value was a corresponding element in the estimated matrix A. For PIDC, the confidence score was used for the ranking, as described above. We did not include SCENIC for the reasons mentioned under the specific method’s description above. For the network methods that identified positive versus negative weights, we took absolute values and ignored the specific effects (see the description for each method).

#### Learning networks using default parameters

Where possible, the default settings in each network method were used to derive a single best final network. For GENIE3, SCODE and PIDC, there are no default parameter settings in the original methods, and weighted scores do not have statistical meanings but only to rank the connections. Therefore, in order to determine the number of edges to be detected in these methods, we set the total number of edges learned to be equivalent or lower to the number detected by the BN method (as a result, the total number of edges in the final network can be less than the BN’s, as some of the edges are eliminated when accounting for directions). TP, TN, FP, FN, precision ($\boldsymbol{P}=\frac{\mathbf{TP}}{\mathbf{TP}+\mathbf{FP}}$), recall ($\boldsymbol{R}=\frac{\mathbf{TP}}{\mathbf{TP}+\mathbf{FN}}$), F_1_ score (${\boldsymbol{F}}_{\mathbf{1}}=\frac{\mathbf{2PR}}{\mathbf{P}+\mathbf{R}}$), False Discovery Rate ($\boldsymbol{FDR}=\frac{\mathbf{FP}}{\mathbf{TN}+\mathbf{FP}}$) were calculated for these learned networks (Additional file 3: Table S1).

#### Principal component analysis (PCA)

PCA was used to investigate the similarity of the learned networks, as measured by the ranking of the interactions inferred. For a given network with *N* nodes, the total number of possible edges is $\frac{\mathbf{N}\left(\mathbf{N}-\mathbf{1}\right)}{\mathbf{2}}$. Each learned network was represented as a vector where each value was the ranking of that interaction in the total network, ranging from 1 to $\frac{\mathbf{N}\left(\mathbf{N}-\mathbf{1}\right)}{\mathbf{2}}$, where 1 corresponds to the top rank. PCA was performed used *prcomp* in R.

#### Comparing networks using characteristics of degree distribution

Degree is defined as the number of edges a particular node has in the network. The degree distributions of the learned networks were compared using the R package *igraph* [43] as another comparison of similarity. Reference networks and the degree distributions of two theoretical network structures were also used in the comparison. An Erdős-Rényi random graph was generated, where the number of genes in each dataset was set as the number of nodes, and the total number of edges was equivalent to the reference network for each dataset. For the scale-free network, we used the Barabási-Albert model [44], and similarly, used the number of genes as the number of nodes for each dataset.

## Results

### Most network inference methods cannot correctly reconstruct networks from simulated gene expression data, including those designed for single cells

**Fig. 2:**
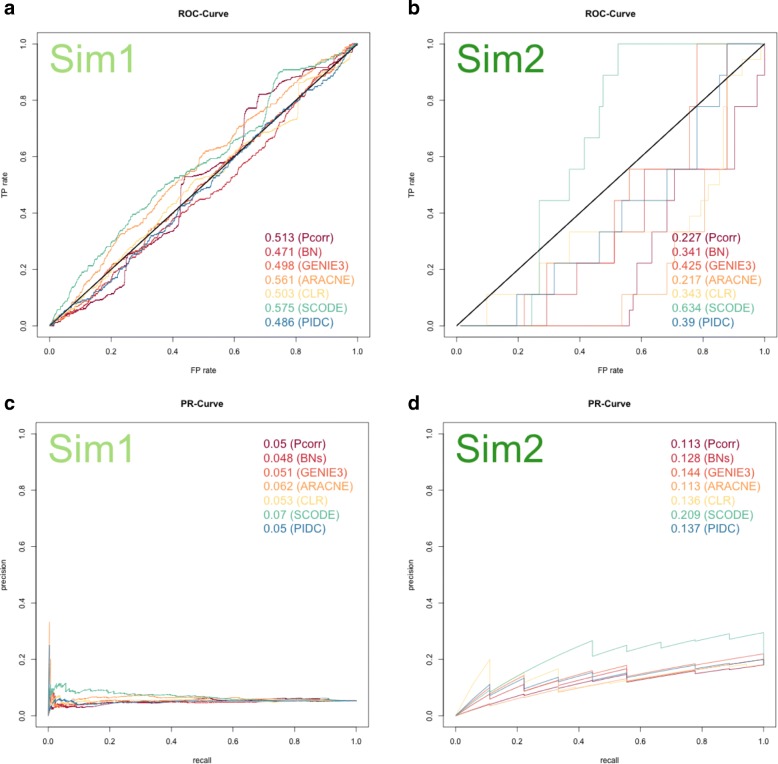
ROC (top) and PR (bottom) curves for each method applied to the simulated datasets. The results obtained from the Sim1 dataset are shown on the left (**a** & **c**) and the Sim2 dataset is shown on the right (**b** & **d**). Diagonal black lines on the ROC curves are baselines indicating the prediction level equivalent to a random guess (**a** & **b**). ROC curves showed that when the threshold changes and more edges are detected, both false positive and true positive rates increased, but the speed of this increase might not be the same. The PR curves show that when the detection thresholds decreased, the number of detected edges increased, with a corresponding increase in recall (more true edges are detected) but decrease in precision (increasing the number of detected edges that are not in the reference network)

Evaluation of the network methods using PR and ROC curves [41] showed that all methods demonstrated poor performance when applied to the simulated datasets that mimic single cell experimental data (Fig. 2). Based on the ROC curves, almost all methods had performance at or around the random baseline (AUC = 0.5) for the Sim1 dataset (Fig. 2a). For the Sim2 dataset, we observed greater diversity in performance across the network methods (Fig. 2b), indicating method specificity in the prediction accuracy. A specific example is SCODE, which had better performance than the other methods and this was consistent for both small and large simulated datasets (i.e., Sim1 and Sim2). However, the AUROC scores for SCODE were 0.575 (Fig. 2a), and 0.634 (Fig. 2b) which despite being the highest for all methods, are still not scores that are indicative of strong performance. Meanwhile, PIDC, which is also a method that was developed for single cell data, did not show a detectable advantage over other methods when applied to either Sim1 or Sim2 datasets, suggesting that all single cell methods do not necessarily perform better than general bulk methods in terms of accuracy, and instead, specific attributes of the method do matter. It can be seen that almost all the methods had high rates of false positives (Fig. 2c and d) even when small numbers of edges were detected (the starting point of the PR curve is 0 on the y-axis for all the methods). This observation indicates that even the edges that were detected with the highest confidence from the simulated single cell dataset were false positives for these methods.

We considered whether it was possible that the lack of performance of all methods was due to the artificial drop-out event that was added to the simulated data. To test this hypothesis, we used the dataset that generated Sim2 without inducing drop-out events (this data is denoted as “Sim2_bulk”, since it resembles the bulk-level simulated data, Additional file 4: Figure S3), and applied the five general methods to reconstruct the network. When we compared these results to those obtained from Sim2 (the single cell simulated dataset), all five methods showed an increase in their AUROC and AUPR scores, although the degree of improvement in performance varied widely. For instance, ARACNE and CLR had AUROC = 0.293 and 0.364, respectively for Sim2_bulk (Additional file 4: Figure S3), which was an improvement over 0.217 and 0.343 from Sim2 (Fig. 2) but qualitatively, did not represent a substantial change. For GENIE3, a much higher score was observed when it was applied to the Sim2_bulk data (AUROC = 0.875, Additional file 4: Figure S3), compared to poor performance when applied to Sim2 (AUROC = 0.425, which is lower than 0.5). Despite the variability in the amount of improvement observed, the improvements were at least consistently observed for all methods. The conclusion from this result is that methods not specifically designed for single cell data have poorer performance when drop-out events are present in the data. Therefore, the poor performance observed is most likely due to the presence of drop-out events. A consistent improvement in performance was not observed for the Sim1 dataset. This is likely due to the fewer number of cell samples in the experimental design of Sim1 compared to Sim2, so that even without drop-out, the sample size is not large enough in Sim1 for the methods to improve their prediction accuracies detectably.

Although ROC curves are the typical choice for comparing classifiers, the PR curve is more relevant for evaluating the network comparison in this situation (Fig. 2c and d). The task of reconstructing a network has a relatively low positive rate (i.e. a sparse prediction problem). In such a problem, the positive predictive value (i.e. precision) is a more useful metric because it measures the proportion of edges detected by the model that is correct, rather than simply the TP (i.e. recall), which is the total number of true edges recovered. Some network methods identify many more edges, and therefore based on TP they may score highly, but upon closer inspection based on precision, the learned network is of lower quality because a lower proportion of those edges are actually true. This is especially relevant when evaluating the learned networks against the PPI reference networks from experimental data. In this situation, the PPIs have been derived from a broader set of cell types, perturbations, and experimental assays that are likely to result in a larger number of interactions. On the other hand, the two experimental datasets selected in our comparison represent highly-specialized cell types and therefore only a subset of the reference networks are expected to be relevant. Therefore, in this case, the evaluation of methods is based on both results from the PR curve and the ROC curve (Fig. 2).

### Similarly, comparisons based on PR and ROC curves reveal poor performance for all methods for reconstructing networks from experimental single gene expression cell data

**Fig. 3:**
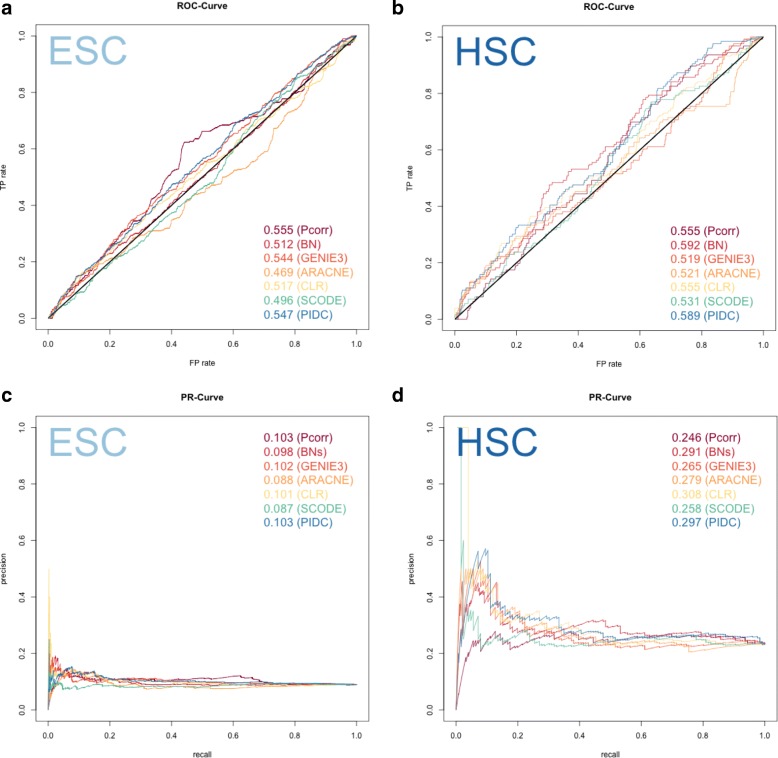
ROC (top) and PR (bottom) curves for each method applied to single cell experimental data. The results obtained from the ESC dataset are shown on the left side (**a** & **c**) and the HSC dataset is shown on the right side (**b** & **d**). Diagonal black lines on the ROC curves are baselines indicating the prediction level equivalent to a random guess (**a** & **b**). In (**a)** and (**b**), almost all the methods aligned with diagonal black lines in the ROC curve, suggesting the predictions are nearly equivalent to a random guess for all these methods. It is easier to observe the behavior of each method in PR curves. Although the ROC curves indicate similar performance across all methods, for the HSC dataset, the PR curve reveal that the methods have different prediction accuracy (when the total number of detected edges is small), as shown when the curve is close to the y-axis, and thus provides another aspect of evaluation

Using the same evaluation framework, all seven network methods were applied to real single cell gene expression data. They demonstrated poor performance, where most of the ROC curves were comparable to the level of random predictions (Fig. 3a and b). Pcorr and BN performed slightly better than other methods for the ESC and HSC datasets, respectively. However, compared to what was observed for the Sim2 dataset (Fig. 2b), their advantages in performance for these datasets were neglible. Similar to the simulated data, almost all the methods had high rates of false positives (Fig. 3c and d) when small numbers of edges were detected. Exceptions for this were CLR and SCODE, when they were applied to HSC data, as shown by the fact that the starting point of PR curve is 1 on the y-axis in Fig. 3d. In this respect, CLR and SCODE had better performance over other network methods, although based on an evaluation using the ROC curve only, CLR did not show any advantage over other methods. In contrast to the simulation data, especially the Sim2 dataset, where methods showed diverse performance (AUROC score ranged from 0.217 to 0.634), neither of the ESC or HSC dataset showed such a range in performance across the network methods (AUROC score ranged from 0.469 to 0.555 for the ESC dataset, and 0.519 to 0.592 for the HSC dataset), suggesting that these methods all consistently give poor performance when applied to real single cell data.

**Fig. 4:**
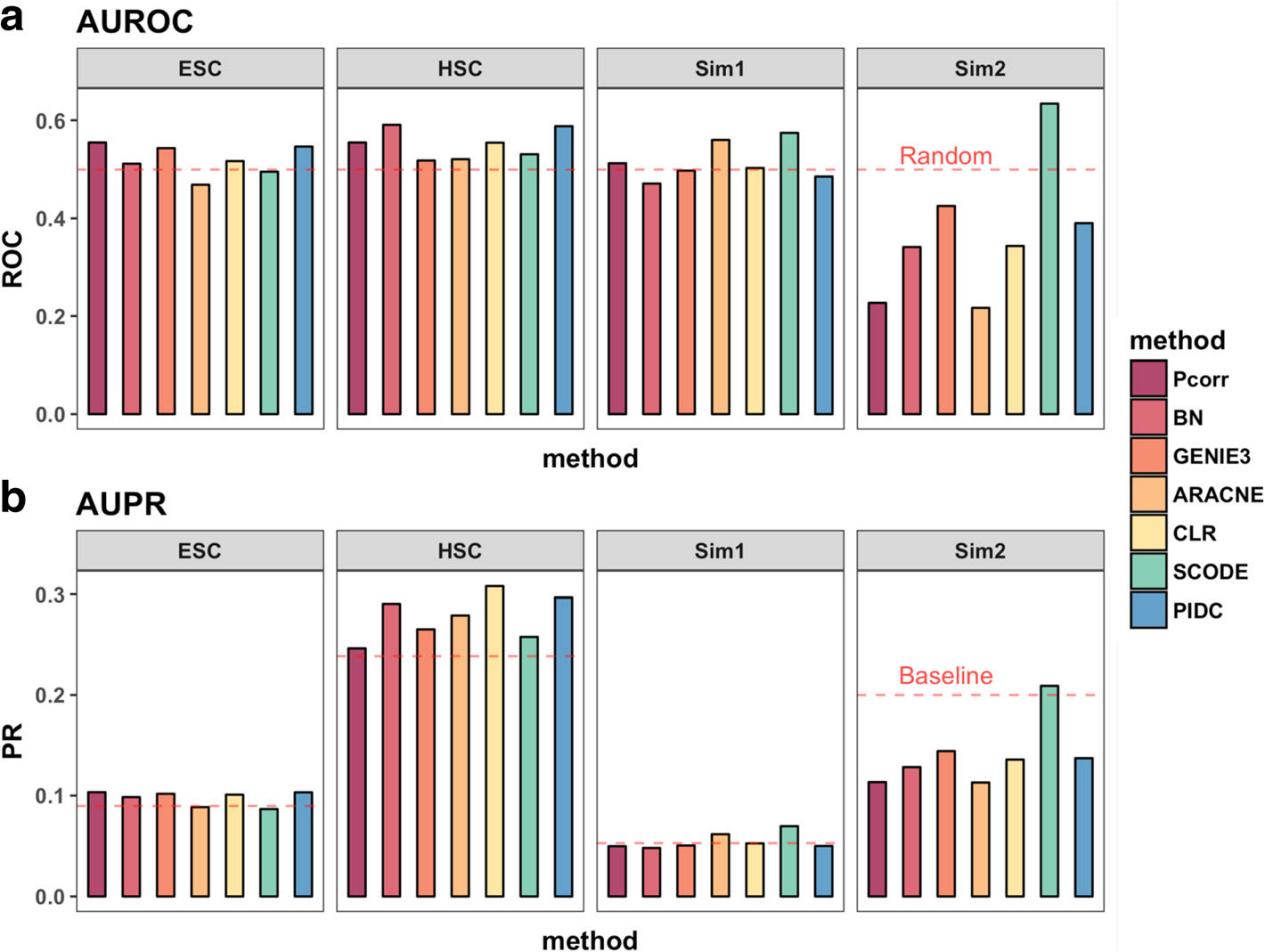
AUROC (top) and AUPR (bottom) scores demonstrate consistently poor performance for most of the methods and datasets. In both panels, the horizontal red lines represent the line of a random guess and baseline, which are the same (= 0.5) for AUROC across datasets (**a**) but differ for AUPR (**b**), since the baseline indicates the value of precision when all the reference edges were recovered, and this depends on the total number of genes in the datasets and the number of edges in the reference networks

Overall performance of the network methods was further assessed by comparing the AUROC and AUPR scores (Fig. 4). Because there are fewer genes in the HSC dataset than the ESC dataset, the number of potential interactions between genes in the network is also smaller. This should result in an easier prediction problem compared to the ESC dataset, which has a larger number of genes (by one order of magnitude). The effect resulting from the differences in sample size for the HSC versus ESC datasets is reflected by the two distinct baselines in the AUPR bar graph (Fig. 4b), and the higher baseline seen in Fig. 3d compared with Fig. 3c.

Based on AUROC and AUPR, the performance of the seven methods (we did not include SCENIC in the evaluation, see Methods) were compared, between and within each dataset (Fig. 4). Using these metrics alone, the simulated data did not score higher than the experimental single cell data when compared to either the HSC or ESC dataset. Moreover, many of the methods had even poorer performance when applied to simulated data. To our surprise, the two simulation datasets seem to be more challenging for most of the network methods to learn from, as demonstrated by the scores that are lower than the random prediction baselines (Figs. 2 and 3). As mentioned earlier, the only exception was SCODE, which obtained a score that was consistently higher than other methods for the simulated data, especially for the Sim2 dataset (Fig. 4a). ARACNE had the second best performance for the Sim1 dataset (behind SCODE), but this performance is not consistent as ARACNE was one of the worst performers when applied to both experimental single cell datasets (Fig. 4).

### The total number of detected edges vary by network method, and when evaluated by a community-based approach, single cell methods have increased prediction accuracy over bulk methods

To understand how many of the same high-confidence interactions were being detected by the eight network methods, we investigated the similarity between detected edges in learned networks using default, “out-of-the-box” settings (with exception for GENIE3, SCODE and PIDC, see Methods). For the four datasets, one network model was generated by each method, resulting in thirty inferred networks in total (SCENIC was not applied to Sim1 and Sim2, see Methods), and a comparative framework similar to the DREAM challenge was applied [41]. We found that the total number of detected edges varied widely from method to method, even when they were applied to the same datasets (Additional file 5: Figure S4). ARACNE and CLR are more likely to detect a higher number of edges compared to other methods, as well as what is captured by the reference networks. In contrast, Pcorr detected the least number of edges when applied to the Sim1 dataset, with zero true positive (Additional file 5: Figure S4C). This might be caused by the low cell sample to gene ratio (100 cell samples to 100 genes), and because Pcorr is based on the significance test of the correlation coefficients, this method is more strongly affected when applied to a dataset with a smaller sample size. In general, the percentage of edges detected by each method had low overlap with those in the reference networks for all network methods. The overlap was slightly higher in general for ARACNE and CLR, but these gains seemed to be negated by the fact that the total number of detected edges for these two methods was consistently higher than all other methods. Especially, for the Sim2 dataset, where ARACNE detected 45 edges in total, resulting in a fully-connected network.

An alternative way to evaluate the overlap between learned networks is to instead, look at how well groups of network methods did at recovering edges from a reference network together. This kind of evaluation is often referred to as a community-based approach [41]. In our study, it was insightful to contrast the accuracy of network reconstruction done by the general, bulk methods combined, versus the single cell methods combined. To test this, we obtained the union of edges detected by the bulk methods, and separately, the union of those edges detected by the single cell methods (Additional file 6: Figure S5). We compared the percentage of true positives relative to the reference networks (the number of edges detected by CLR and ARACNE were too large to make meaningful comparisons, therefore we did not include them in this analysis). We included the edges that were detected by any of Pcorr, BN, or GENIE3 for bulk methods, and included those that were detected by any from SCENIC, SCODE, or PIDC (for Sim1 and Sim2, only SCODE and PIDC) for the single cell methods.

Except for the ESC data, the community-based approach by single cell methods had better prediction performance compared to the general bulk methods. This might be caused by the fact that the more recent single cell network methods have more diversity in their inference algorithms, while the general methods we included in this analysis are somewhat similar as they are mostly based on regression models. For instance, the three bulk methods are made up of Pcorr, BN, and GENIE3 where Pcorr is based on partial correlation, and both BN and GENIE3 use regression modeling for edge prediction; these are two statistical approaches that draw a large degree of overlap. Alternatively, the single cell methods collectively represent divergent approaches where SCENIC is based on a co-expression network combined with bioinformatics knowledge, SCODE uses ODEs, and PIDC is a MI-based method. Therefore, each single cell method learned a network that was distinct, and combining the results of the single cell networks intuitively may give rise to a more comprehensive overlap with the reference networks.

### A common set of edges are detected by different methods but a large number of method-specific and data-specific edges are observed for both experimental data and simulation data

**Fig. 5:**
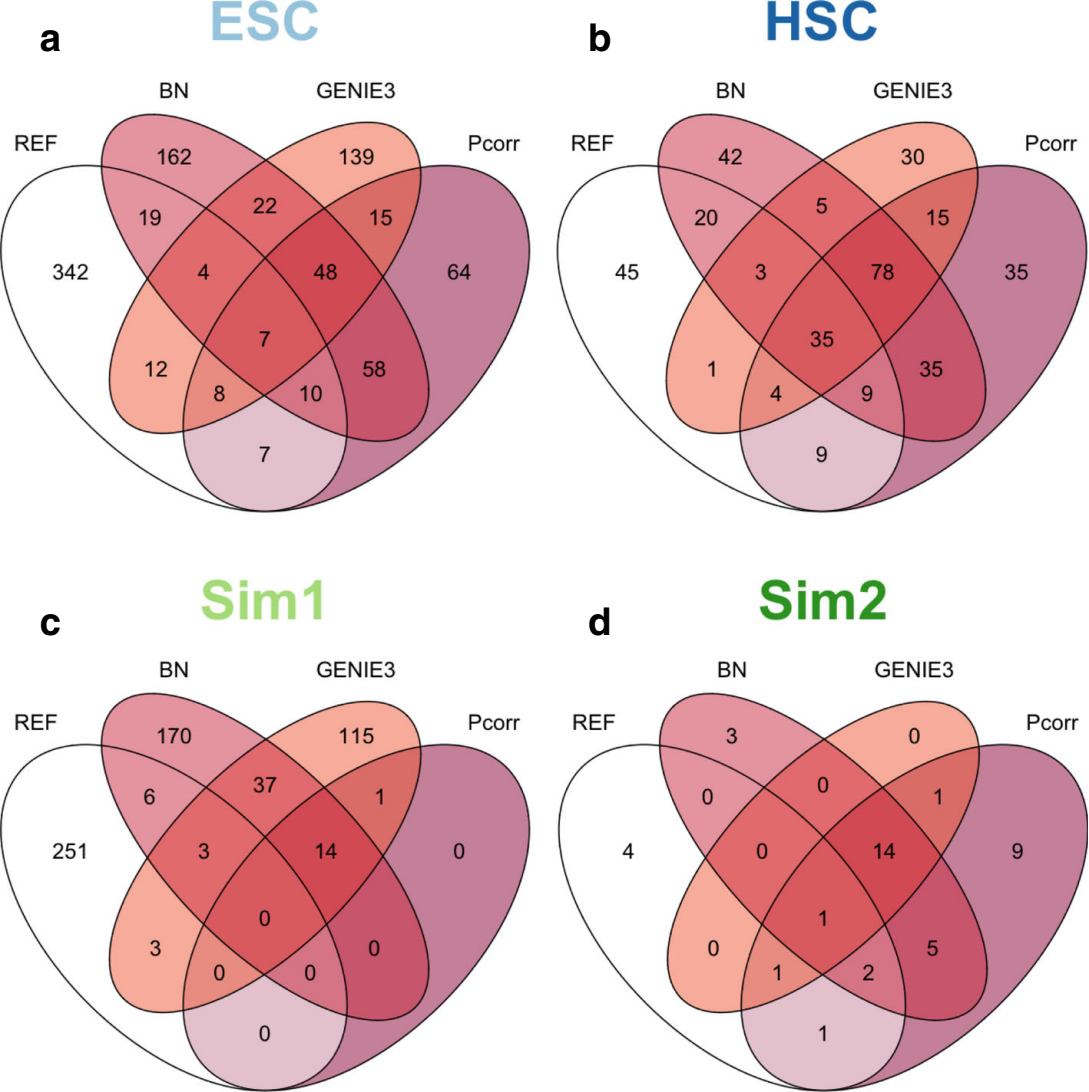
Intersection of reconstructed networks from general methods and reference networks outlines the ability of the different methods to identify the same true positives. These methods detected a core set of interactions in the learned networks for the ESC data (**a**), HSC data (**b**), and two simulated datasets, Sim1 (**c**) and Sim2 (**d**). In general, each method also detected edges that were unique to the method and the dataset, except for Pcorr in Sim1 (**c**, see text). Only a small set of edges in the reference networks were recovered by intersection of three methods, and also different methods detected edges that were unique. Moreover, we show that even after combining all the methods, there were still edges in the reference network that were not detected, as indicated in the white section for each panel

**Fig. 6:**
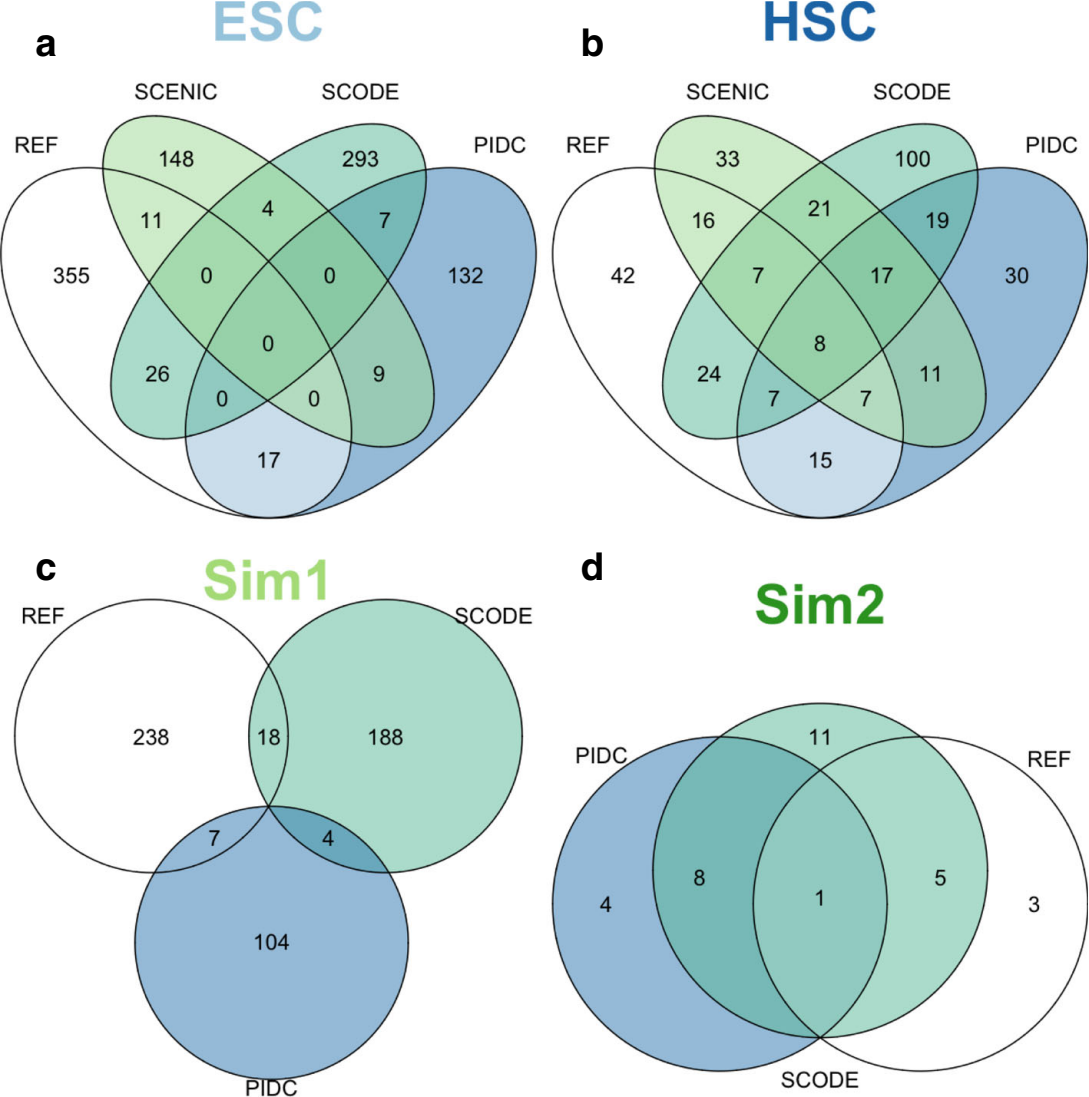
Intersection of learned networks from single cell methods and the reference network highlights the differences between the edges that are uniquely detected by each method. Although single cell methods detected a common set of interactions, there was far more inconsistency in their detections, compared to the results of general methods. Each method, however, was able to detect some ‘correct’ edges that are unique to each method (as indicated from the overlap between each colored ellipse and the white ellipse, also see Results). Similarly, only a handful of edges were commonly detected by all three methods for the HSC and Sim2 datasets (**b** & **d**), and no single edge was commonly detected for the ESC and Sim1 datasets (**a** & **c**)

Given the large variation in the number of edges detected by the network methods, we next compared the overlap in edges to investigate whether a core set of edges was detected (Figs. 5 and 6), and how many of the detected edges were also in the reference network. For the five general network methods in our study, only results from Pcorr, BN and GENIE3 are shown, for the same reasons mentioned above. It is clear that some of the edges were consistently detected, no matter which methods were used, e.g. 48 + 7 common edges for the ESC dataset and 78 + 35 common edges for the HSC dataset were detected by these three general methods (Figs. 5a and b). This was observed in both real single cell experimental data and simulated data, although networks for the experimental data showed more common edges (e.g., 48 + 7 common edges were commonly detected for ESC, amongst 96 genes, while only 14 + 0 edges were consistently detected for Sim1, amongst 100 genes). Meanwhile, a subset of edges that were unique to a specific method was also observed. For instance, for the HSC dataset, 42 + 20 edges were detected by the BN only, and not detected either by Pcorr or GENIE3. An exception for this was when Pcorr was applied to the Sim1 dataset where all edges (15 in total) were also detected by GENIE3, and 14 out of these 15 were detected by the BN. It is worthwhile noting that the total amount of detected edges by Pcorr was much smaller for this dataset. The high degree of overlap between these methods was not surprising, since they are dependent on the same mathematical rationale which involves a regression model, as mentioned above.

We also compared the quality of detected edges by examining the overlap between multiple network methods and the reference networks (Fig. 5, indicated by the number on the center of each Venn diagram). We found that not all edges were able to be recovered by the reconstructed networks, regardless of which datasets were used. In fact, the number of edges in the reference networks that were recovered from all three methods is far lower than the total numbers of edges in the reference networks. For instance, for the ESC dataset, 7 “true” edges were recovered by all the methods while there were 409 total “true” edges in reference network (Fig. 5a). For the simulation datasets, we found that the recovery rate of reference edges was also poor. For instance, amongst 14 edges that were commonly detected from the Sim1 dataset using three general methods, none of them were in the reference network (although this might be affected by the fact that the number of true positives for the Pcorr method was zero).

For all three single cell methods (SCENIC, SCODE and PIDC, with SCENIC only applied to ESC and HSC dataset, see Methods), we found that there were fewer edges overlapping each of the learned networks, and the recovery of reference edges was even poorer (Fig. 6). For instance, there were no edges detected consistently by all three methods for the ESC dataset. For the Sim1 dataset, there were only 4 edges detected by both SCODE and PIDC, but none of them were in the reference network. What was interesting is, in the ESC dataset, ‘correctly’ detected edges were unique to each method (Fig. 6a). For instance, SCENIC detected 11 edges that were present in the reference network, but none of these 11 edges were detected from either SCODE or PIDC. This was the same for SCODE (26 ‘correct’ edges detected), and PIDC (17 ‘correct’ edges detected). This may explain why there was such low consistency amongst the edges identified by the methods, and the low prediction accuracy when their intersect was examined. That is, these single cell methods utilize completely different approaches, which did not reduce their performance largely by themselves, but did reduce their prediction accuracy when intersected edges were examined. This also supports the possible explanation observed when evaluating the methods based on the community-based approach (Additional file 6: Figure S5), where a better prediction accuracy was seen by the union of single cell methods, than the general methods for three out of four datasets. This result suggests that single cell methods were able to detect ‘correct’ edges in a unique manner, which also suggests the importance of a community-based method for better overall network prediction.

It is possible that this inconsistency in overlap may be due to the fact that the reference network does not accurately reflect the true GRN controlling the cells of interest in this comparative study. This can be understood by considering that the PPIs used to make up the reference networks were derived from a variety of information sources that reflect a diversity of experimental designs, cell types, and developmental stages that were different from the variables in our study. This therefore represents a significant limitation to those results that are based on comparisons with the reference network because it represents a much broader GRN whereas the two datasets were generated from stem cell populations, and likely to be governed by a more specific set of regulatory interactions. It is worth highlighting that the reference network was derived mainly from genome-wide assays whereas the Fluidigm datasets represent only limited numbers of genes that were profiled.

Also, 48 and 78 potential interactions in the ESC and HSC datasets respectively, were detected by all general methods, and 17 potential interactions in the HSC were detected by all single cell methods. These edges, however, did not exist in the reference network and therefore would be automatically discounted as false positives. This result suggests that some indirect interactions are consistently detected by these methods, or some interactions genuinely exist in these datasets but have not yet been observed in the reference networks, especially for the single cell experiments. We also acknowledge that the fact that the overlap between the three methods was larger for the HSC dataset than the ESC dataset, may be due to the larger sample size for the HSC dataset, which may give rise to a more consistent, stable prediction problem by the different network methods.

### Investigating the similarity of interactions show that there is no consistent clustering of any of the learned networks from the different methods

**Fig. 7:**
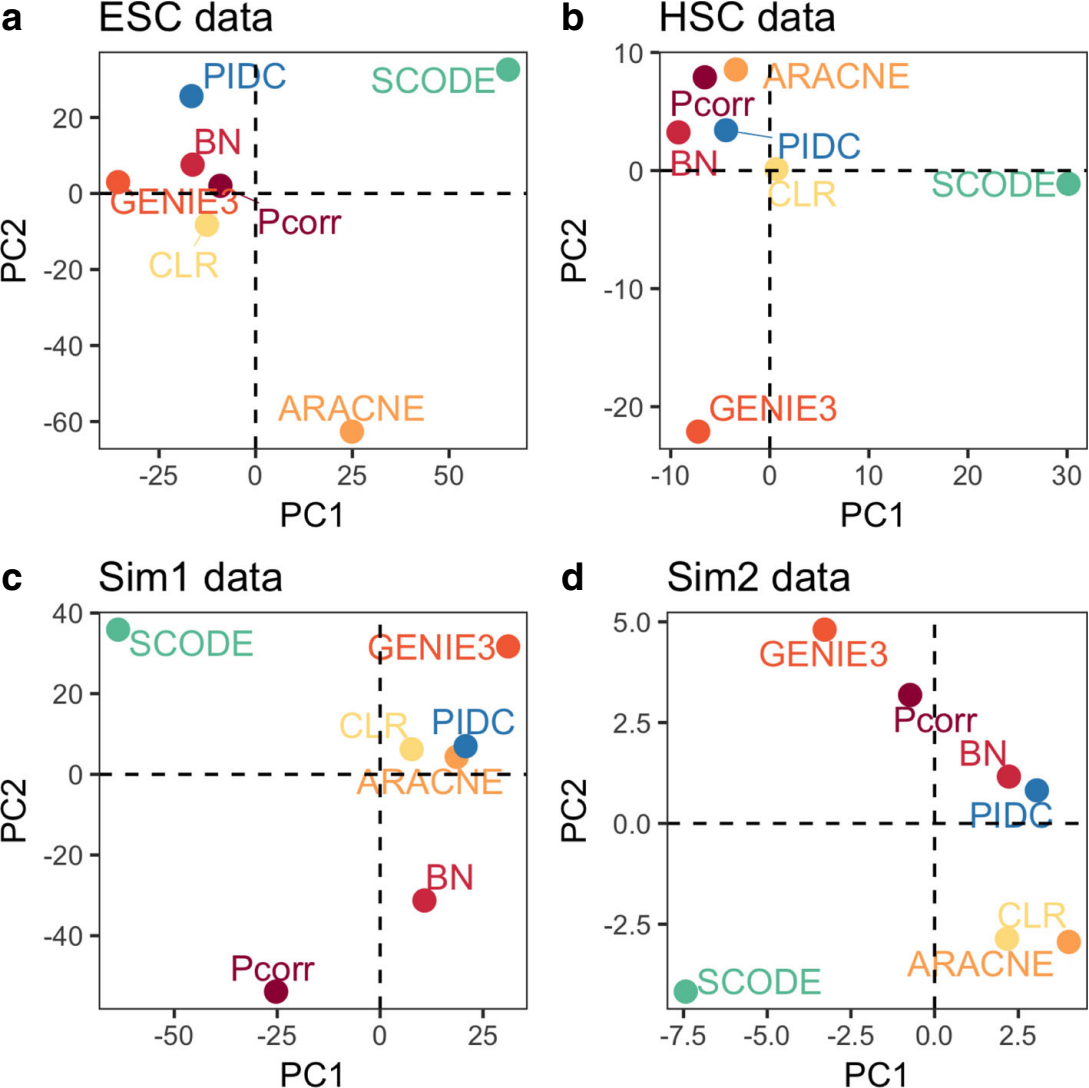
Investigating the similarity of the networks produced by the seven network methods**.** The PCA plots indicate how much each method is similar to each other in terms of edge detection ranking. We show that there is no consistency in the clustering of these methods, and any similarities amongst them vary based on the datasets. SCODE was notably a consistent outlier amongst all the methods

To gain a clearer understanding of which methods may infer networks with greater topological similarity, we applied Principal Component Analysis (PCA) to assess network similarity for each of the datasets. The rationale of this analysis is to investigate whether methods that are based on similar principles might infer networks with similar edge detection patterns, which is measured by the rankings based on the edge weights. For instance, since ARACNE, CLR and PIDC are methods based on measuring MI, we wanted to test if they all assigned higher weights to the same set of edges and reconstruct networks with similar structures. To conduct this comparison, a vector of the length equal to the number of potential interactions with *N* nodes, ($\frac{\mathrm{N}\left(\mathrm{N}-1\right)}{2}$) was assigned with ranks based on the possibility to be detected using that method (see Methods). This analysis was based only on the learned networks, and was independent of the reference networks. No significant clusters were apparent in the PCA plots for any of the four datasets (Fig. 7). For methods such as ARACNE and CLR, where we expected the learned networks to be similar to each other, these did not always correspond to the closest points to each other in PCA-space in the four plots (Fig. 7). SCODE, which was the best performer for the simulated datasets, has totally different network prediction pattern compared to all others. This result provides further evidence that despite similarity in mathematical rationale, the performance of ARACNE and CLR are data-specific.

### The degree distributions of the reconstructed networks show different underlying features based on known topologies from the single cell data

Another approach to evaluating similarity in network topologies is to compare the degree distributions to known theoretical graph structures (Additional file 7: Figure S6). We compared the degree distributions obtained from the learned networks and the reference network, and to two theoretical distributions, the random graph, and the scale-free topology network. Scale-free topology is a network structure that has features of heavy tails and peaks at the point close to zero. In addition to social networks, many large biological networks have been reported to follow a scale-free topology structure. A random graph is another type of simple network structure where the degree distribution follows a Bernoulli distribution. We found that in the simulated data, the reference network was in close agreement with the theoretical network for scale-free topologies (Additional file 7: Figure S6C and D). The consistency between scale-free topology and the reference network can be explained by the fact that the simulation datasets were subtracted from a real biological network of *E. coli*, which might have a structure that follows the scale-free topology networks. By contrast, for the HSC and ESC datasets (Additional file 7: Figure S6A and B), the reference networks did not overlap with either of these two theoretical distributions, and instead represented a mixture of the two features. For all four datasets, the BN was more likely to learn networks with higher degree, compared to other methods, and resembled the random network structures. This result further supports the finding that the underlying network topologies are different for the reference network and networks learned from single cell data, suggesting that more optimal reference-based networks need to be generated and made available for more reliable evaluation of network performance.

## Discussion

This study investigated whether any of the five commonly-used network methods were suitable for reconstructing networks from single cell gene expression data, and if three newly-developed single cell specific network methods would have better performance for predictions. Evaluating the quality of the reconstructed network is challenging due to the fact that inferring networks is a computationally demanding task to which no single solution exists and therefore there are many ad hoc variations on how to identify network interactions. The evaluation is further complicated by the fact that in genetics, regulatory networks are never identified comprehensively, and therefore the benchmarks that are used to evaluate the reconstructed network are always inherently incomplete. Moreover, the reference networks we do have are obtained through a host of experimental variables, e.g. technology platforms, cell lines, primary cell types, environmental conditions, and other factors, that most certainly affect the relevance or specificity for subsets of interactions in the reference networks. Until we have amassed enough data to resolve the contributions of these different factors definitively, any comparison to reference networks is only ever an approximation under the best circumstances. To offset the limitations associated with the reference networks, it was necessary to not rely solely on this comparison to conduct the evaluation. Therefore, it was important to generate in silico data from known networks as an additional way to evaluate the network reconstruction methods for single cell data.

The results from the DREAM5 network prediction competition [41] suggested that methods for inferring GRNs are specific to certain types of data, and there was no one stand-out method that performed the strongest for all types of data. In our study, similar trends were observed where we concluded that there was no one method that performed significantly better than any of the others for all of the datasets under the comparisons conducted. Rather, more significantly, most of the methods in our evaluation performed relatively poorly for all datasets, at a level that was close to, if not worse than, a random guess. Overall, these results suggest that the reconstruction of networks from single cell data is not reliable or accurate for most of methods tested.

The inability to construct networks reliably for single cell gene expression data may be the result of the distinct features associated with single cell data that make it more challenging with existing network methods. Assumptions underlying five general methods (e.g. Normality distributions of the data) may not be suitable for single cell expression data. For single cell network methods, one method may only be able to identify particular interactions between the genes, while neglecting others. The poor performance could also be the result of the fact that reference networks are not truly representative of relevant interactions occurring in single cell biology. For single cell experimental data, the true network is unknown, and a protein-protein association database obtained from previous research does not necessarily reflect the true scenario in single cells. Therefore there might be a large number of false positives in the reference network itself, i.e., no genuine interaction is observed in the true single cell state, even though it may have been detected in previous experimental conditions and therefore is present in the reference network. These possible reasons all support a rationale for further investigation into the development of network methods that are specifically optimized for single cell gene expression data, as well as access to more accurate resources for network evaluation and validation.

As mentioned, evaluating the performance of each method using simulated datasets is a straightforward process, since we know the underlying network. By contrast, finding a clear benchmark to evaluate performance for single cell experimental data is harder since a genuine “gold standard” reference does not yet exist. In this study, we tried to incorporate a “bronze standard” in order to compare the network methods but it is critically important for a control reference network for single cells to be generated for the field to move forward. The reference networks were extracted from the STRING database, where any gene pair was included as an edge in the reference networks and not limited to single cells or the HSCs, ESCs or cell types that represented a specific data set of interest. In this way, it might be expected that a larger number of false negatives and true positives are seen from the learned networks than the true case, and overall the use of the PPIs as a reference network may be insufficient as an accurate benchmark for network reconstruction.

Because of the limitations associated with the reference network, we placed more emphasis on the precision of each method in the network comparisons. Specifically, we were interested in assessing how many of the edges that were detected were actually in the reference network (i.e. reported before in the literature), rather than detecting what percentage of the interactions in the reference networks were recovered. Hence, the use of the PR curves was a more appropriate metric for our evaluation. Additionally, another advantage of using PR curves is that they can more readily reflect distinct features of the data. For instance, the major difference between Sim1 vs. Sim2, as well as ESC vs. HSC, is the ratio between sample size and the number of genes in the dataset. In Sim1 datasets, the total number of genes is 100, and hence the number of potential edges is 4950, or 100 choose 2. Therefore, the ratio between the number of true connections in the reference network and the total combination of two nodes is lower than those of Sim2, which is 45, or 10 choose 2, suggesting that there may be difficulties in general for making predictions with so many options. This can be seen in the baseline level of PR curve that is far lower for ESC and Sim1, than the baseline for HSC and Sim2 (Figs. 2c and d, 3c and d). This trend is masked in the ROC curves and cannot readily be identified. For the ROC curves, the diagonal line reflects the baseline, but it is clearly the same for all datasets (Figs. 2a and b, 3a and b) irrespective of the underlying experimental design.

Of note, GENIE3 showed far better performance than other general methods for the Sim2 dataset in the case when artificial drop-out was removed from the simulated data (Additional file 4: Figure S3). This strong performance observed for ‘bulk’ simulated data was consistent with the previous results from the DREAM challenges, where GENIE3 was the best performer on the in silico data [31]. Therefore, in both the DREAM challenge and this study, it is clear that GENIE3 is a suitable approach for the simulated datasets, when bulk sample gene expression data is considered for network reconstruction. In our comparison, however, with induced drop-out included, the performance of GENIE3 was severely affected in ways that were apparent for the Sim2 dataset, where the AUROC score fell below 0.5 (AUROC = 0.425, Fig. 2). This is not surprising, given the fact that methods like GENIE3, although robust for bulk sample gene expression datasets, were not specifically designed for datasets with such high rates of zero values, as in the case of single cell data. For Sim1 data, the difference between ‘bulk’ and ‘single cell’ data are less significant, probably because the sample size affected the performance of these methods. This further highlights the importance of using data generated by large sample sizes to derive the most accurate network inference possible.

Out of all the methods, SCODE obtained the highest score in general for the simulated datasets (ranked as the best performer for both Sim1 and Sim2, Figs. 2 and 4). Significantly, we found that SCODE did not perform well when applied to single cell experimental data. This result further emphasizes the data-specific performance for each method, and that the utility of the methods depends on the study of interest. In particular, the strong performance of SCODE for simulated data was not surprising, considering the similarity between the mechanism in which the simulated data was generated using GNW, and the prediction algorithm used in SCODE (i.e., pseudotime is inferred from the static single cell data, and then the algorithm uses ODEs to describe the molecular dynamics based on the inferred time series data). In addition, it also highlights the discrepancy between using simulation data to study the performance of network prediction, versus using real single cell experimental data. Multiple reasons may exist to explain this discrepancy, but the most relevant reason might be that there are fundamental differences in the structure of single cell data that cannot yet be captured by simulated data. This is evident by considering the data distributions that vary between experimental data and simulated data, even after we imitate the simulated data to real data by inducing drop-out (Additional file 2: Figure S2).

The performance of the BNs was not stronger than any of the other methods, except when it was applied to the HSC dataset. BNs are known to require large sample sizes to accurately learn the model structure. The results from our comparative study support this observation because HSC has the largest number of cells (3934 cells) amongst the four datasets used in our comparison, and the BN was the best performer amongst all methods when applied to the HSC dataset. Although the Sim2 dataset (sample size of 1000 cells) was generated to mimic the sample-gene ratio of the HSC dataset, again, without a clearer understanding of the observed differences in the performance for real data and simulated data, it is hard to explain concretely why the BN method performed better for the HSC dataset but not the Sim2 dataset. Another explanation for the poor performance of BNs compared to other methods may also be due to the fact that BNs do not allow self-loops, which prevents BNs from predicting interactions that are genuinely represented by this network structure. These limitations further highlight the need to consider methods that are appropriate to the study of interest. Assessment based only on prediction performance of the learned network may not be an adequate test to fully understand the utility of the network method.

In this study, we did not limit the parameter for the number of edges detected when using ‘out-of-the-box’ setting network reconstructions. By doing so, we designed the study to be able to compare the most straightforward networks that can be generated from ‘default’ settings of the algorithms, as these are most likely to be produced by a typical user. At the same time, there are two potential limitations that should be considered when comparing the results from this study. First, some of the network methods do not have internal thresholds to determine the number of edges, in which case we had to specify a number and therefore used the number of edges predicted from the BN, as the maximum number, for easier comparisons. Second, for some methods, the ‘default’ parameter was not necessarily a good choice, which was made clear by the fact that for the Sim2 dataset, ARACNE reconstructed a fully-connected network. An alternative approach to overcome these limitations, and potentially facilitate an easier comparison, is to fix the total number of predicted edges, so that all the reconstructed networks would have the same number of edges. However, a critical question that this may raise is, how the fixed number of edges can be defined given a particular number of cell samples and genes? An ad hoc number is likely to bias the results, by providing a setting that allows for stronger performance by some methods over others. The rationale for this can be seen from the diversity observed in the number of edges detected by the different methods (Additional file 5: Figure S4). Moreover, it is unrealistic that the number of expected edges is known a priori for the network, and therefore a comparison done with fixed numbers for all methods does not seem feasible.

The decision to include BNs in the network methods was motivated by the fact that BNs represent interactions between genes as probabilistic events. The rationale is that if BNs could learn the GRN accurately, then it may be more informative to use conditional probabilities as a way to model single cell gene expression data, given the digital nature in which transcripts are produced. BNs also allow for inferring directed interactions, and therefore may be more accurate and useful for perturbation studies involving single cells, such as knock-out or knock-in experiments, which were not addressed in our study. Overall, modeling GRNs as probabilistic interactions allows for multiple network representations for the one single cell dataset. This is a new paradigm for network biology, where traditionally, it is thought that one GRN represents a cellular phenotype. Given the stochasticity of gene expression in single cells, it is not implausible to consider one GRN with multiple conditional probabilities that explains the variability or heterogeneity of gene expression by accounting for different states that interactions between genes may operate through. Although BNs did not have strong performance in this study, given the mathematical framework of BNs, it seems likely that modifications to this specific kind of network approach may yield new methods to model single cell gene regulatory networks in ways that allow us to gain deeper insight into how GRNs are controlled at the level of single cells.

When considering the mathematical rationale underlying the current set of single cell network methods, SCENIC is similar to GENIE3, PIDC is similar to the MI-based methods ARACNE and CLR, and SCODE is similar to Pcorr and BN through its relationship to regression modeling. But the probabilistic aspect of BNs is not well-represented by any of the single cell methods currently available. While standard BN software has previously been applied to single cell data, such as BANJO for single cell RNA-sequencing data [45], there are many opportunities in which steps within the BN method could be modified for single cells. For instance, there are two main stages of BN learning, the first being the structural learning step, and the second is the parameter learning step. The latter is where probabilistic interactions are inferred, and this is specifically where methodological innovations could be applied to be directly relevant to single cell gene expression data. In our study, we have used only one implementation of the BN, as a means to conduct comparisons with other network methods, but a potential source of technological advances exist for BNs for this specialized problem of network reconstruction [46].

## Conclusions

In this study, we performed a comparison of eight network algorithms to test their ability to reconstruct networks from single cell gene expression data. In particular, we included both general, popular network methods that were developed for bulk samples, as well as three single cell-specific methods that were developed recently. We found that single cell network inference methods do not necessarily have better performance than general methods, and even for the method that performed well for simulated data (SCODE), it did not have a higher prediction accuracy than other methods for the experimental single cell data. In addition, we found that when applied to simulated data without drop-out, GENIE3 was the best performer amongst all general methods, while this did not generalize to the simulated ‘single cell’ data where drop-out was induced. From this study, we therefore conclude that for single cell data, either generated from experiments or simulations, these networks methods had consistently poor performance for reconstructing networks.

Not surprisingly, the networks reconstructed from these methods had features that were distinct from each other. The two general methods using mutual information detected far more edges in the network than methods based on other approaches. Community-based detection accuracy was higher for single cell methods, than general bulk sample methods, and the edges identified by each of the methods from the same datasets were different. Assessment of the overlap of the detected edges indicate that most of the methods can learn edges that are specific to the method. Lastly, the PCA analysis shows that SCODE had a distinct pattern of prediction, while even the presumably similar methods (e.g., ARACNE and CLR are considered to be similar) did not consistently cluster together in the plots, indicating that their network topology was not necessarily more similar to each other than the other methods.

For the first time, this study provided a comprehensive assessment of the five common network reconstruction algorithms, as well as three new single cell network reconstruction algorithms, for single cell gene expression data. Reconstructing GRNs from gene expression data has been one of the most important topics in systems biology. Therefore, evaluating the performance of existing algorithms and understanding their limitations when applied to a newer wave of data, such as single cell gene expression data, is extremely helpful to facilitate further development of new methodologies that are specific for single cells. Such models would further provide new opportunities to understand cell-to-cell heterogeneity, and other biologically interesting questions such as stem cell differentiation and cancer development.

## Additional files

Additional file 1: **Figure S1.** Simulation parameters using GNW to generate simulated datasets, Sim1 (top) and Sim2 (bottom). For Sim1, we sampled 101 times points (the first time point at *t* = 0 was not used) from a series of time series data, with other parameters kept the same as the ones used in the DREAM4 challenge, and eventually obtained S = 100. For Sim2, we sampled 11 time points (the first time point at t = 0 was not used) from 100 series of time series data, with the other parameters kept the same as the ones used in the DREAM4 challenge, and eventually obtained S = 1000. (PDF 347 kb)

Additional file 2: **Figure S2.** Data distributions of the single cell experimental and simulated datasets. The two experimental datasets showed evidence of zero-inflation which is a typical feature of single cell gene expression data (A & B). For the simulated datasets (C & D), zero values were added according to a probablistic scheme for drop-out events as per experimental single cell data. The simulated datasets (C & D) still have notable differences in the data distributions compared to the real single cell experiment data (A & B), despite the underlying mechanisms designed to mimic features of the single cell data. (PDF 383 kb)

Additional file 3: **Table S1.** Summary of metrics used to evaluate performance of each method. (DOCX 19 kb)

Additional file 4: **Figure S3.** ROC (top) and PR (bottom) curves for general methods applied to the Sim2 datasets without the drop-out effect. To examine whether poor performance observed was due to the methods, or the complexity of the datasets, we used the Sim2_bulk dataset (Sim2 without inducing drop-out). From both AUROC and AUPR scores, all five methods had improved performance on the Sim2_bulk dataset compared to the performance on Sim2 data where drop-out was included. (PDF 180 kb)

Additional file 5: **Figure S4.** Variable numbers of edges (and true positives) were detected for each method from the four datasets. Each bar represents the total number of edges detected (colored bar) and the number of True Positives (TPs) among them (white bar) when using the default setting of each method to apply for each dataset. The total number of edges detected varies widely for each method, and ARACNE and CLR detected far more edges than the reference networks and other methods. For the Sim2 dataset, ARACNE recovered a fully-connected network. (PDF 272 kb)

Additional file 6: **Figure S5.** Comparison of community-detected network by bulk sample network methods versus single cell network methods. For general methods (or ‘bulk’ methods), we obtained the union of edges from Pcorr, BN and GENIE3, and compared how this union network overlaps with the reference network. For single cell methods, we applied the same comparison by obtaining the union of SCENIC (included only for the ESC and HSC data), SCODE and PIDC. For three out of the four datasets, the union of the single cell methods had a higher recovery rate than the union of general methods. (PDF 194 kb)

Additional file 7: **Figure S6.** Degree distributions of the learned networks (from Pcorr, BN, GENIE3, SCODE and PIDC), reference networks, and theoretical distributions of random graph and scale-free network. The network models were learned using default settings, and degree distributions were represented as density plots. Theoretical models of random graphs and scale-free network were generated for each dataset. For the simulated datasets, reference networks overlapped relatively well with networks with the theorectical scale-free network (C & D), while for single cell datasets, the reference networks had much wider distributions. (PDF 745 kb)

## Abbreviations

ARACNE: Algorithm for the Reconstruction of Accurate Cellular Networks
AUC: Area under the curve
AUPR: Area under the PR curve
AUROC: Area under the ROC curve
BIC: Bayesian Information Criterion
BN: Bayesian network
CLR: Context Likelihood of Relatedness
DPI: Data Processing Inequality
*E. coli*: *Escherichia coli*
ESC: Embryonic stem cell
FPR: False positive rate
GENIE3: GEne Network Inference with Ensemble of Trees
GNW: GeneNetWeaver
GRN: Gene regulatory network
HSC: Hematopoietic stem cell
MI: Mutual Information
ODE: Ordinary Differential Equation
PCA: Principal component analysis
Pcorr: Partial correlation
PID: Partial Information Decomposition
PIDC: Partial Information Decomposition and Context
PPI: Protein-protein interaction
PR: Precision-recall
ROC: Receiver operating characteristic
SCENIC: Single-Cell rEgulatory Network Inference and Clustering
SCNS: Singe-cell network synthesis
Sim1: Simulation 1 (see Table 1)
Sim2: Simulation 2 (see Table 1)
TPR: True positive rate
