# Supplementary material for: Evaluating methods of inferring gene regulatory networks highlights their lack of performance for single cell gene expression data
Source: BMC Bioinformatics. 2018 Jun 19;19:232. doi: 10.1186/s12859-018-2217-z (PMC6006753; doi:10.1186/s12859-018-2217-z)
Supplement: Supplementary file 1 — Figure S1. Simulation parameters using GNW to generate simulated datasets, Sim1 (top) and Sim2 (bottom). For Sim1, we sampled 101 times points (the first time point at t = 0 was not used) from a series of time series data, with other parameters kept the same as the ones used in the DREAM4 challenge, and eventually obtained S = 100. For Sim2, we sampled 11 time points (the first time point at t = 0 was not used) from 100 series of time series data, with the other parameters kept the same as the ones used in the DREAM4 challenge, and eventually obtained S = 1000. (PDF 347 kb) [file 12859_2018_2217_MOESM1_ESM.pdf]

Figure S1

A

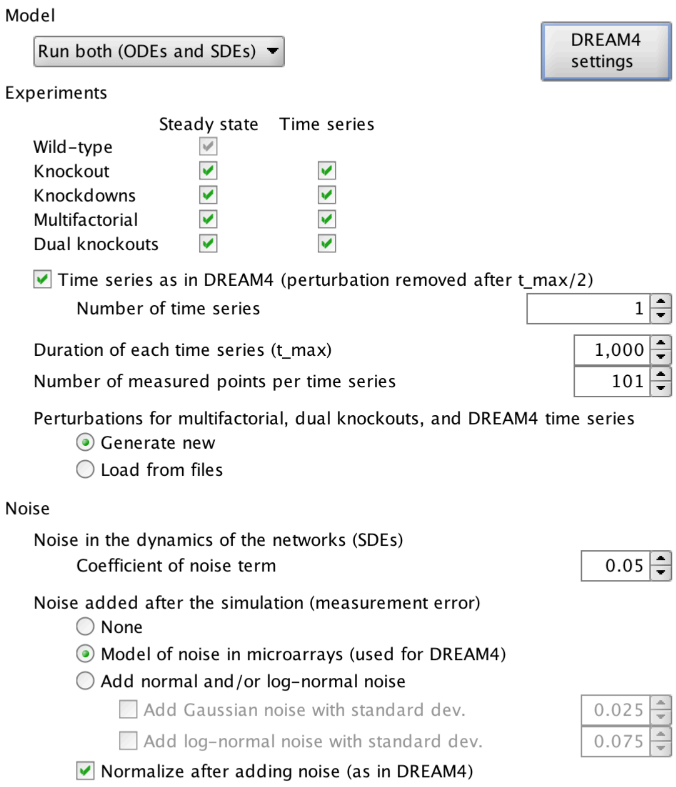
Model
Run both (ODEs and SDEs)
DREAM4 settings
Experiments
Steady state
Time series
Wild-type
Knockout
Knockdowns
Multifactorial
Dual knockouts
Time series as in DREAM4 (perturbation removed after t_max/2)
Number of time series
1
Duration of each time series (t_max)
1,000
Number of measured points per time series
101
Perturbations for multifactorial, dual knockouts, and DREAM4 time series
Generate new
Load from files
Noise
Noise in the dynamics of the networks (SDEs)
Coefficient of noise term
0.05
Noise added after the simulation (measurement error)
None
Model of noise in microarrays (used for DREAM4)
Add normal and/or log-normal noise
Add Gaussian noise with standard dev.
0.025
Add log-normal noise with standard dev.
0.075
Normalize after adding noise (as in DREAM4)

B

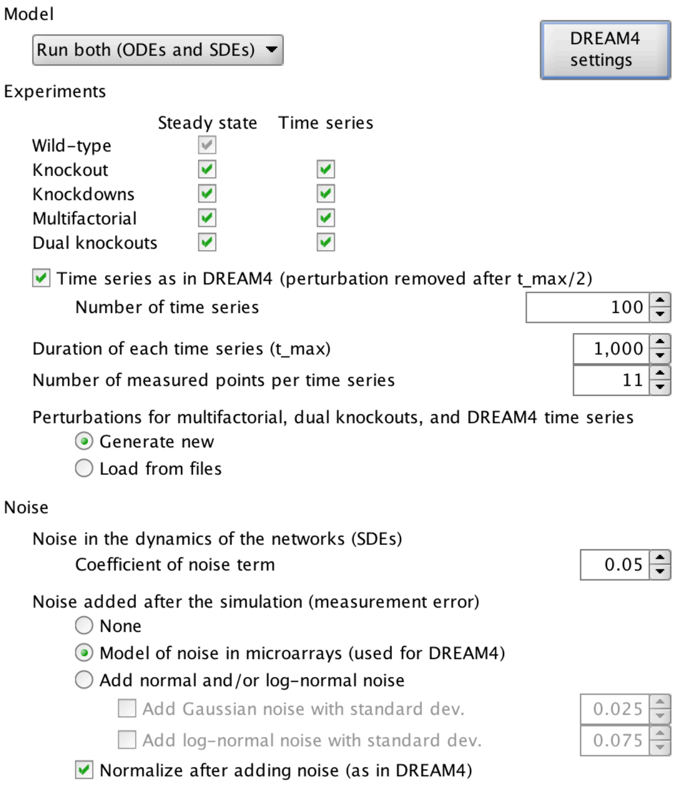
Model
Run both (ODEs and SDEs)
DREAM4 settings
Experiments
Steady state
Time series
Wild-type
Knockout
Knockdowns
Multifactorial
Dual knockouts
Time series as in DREAM4 (perturbation removed after t_max/2)
Number of time series
100
Duration of each time series (t_max)
1,000
Number of measured points per time series
11
Perturbations for multifactorial, dual knockouts, and DREAM4 time series
Generate new
Load from files
Noise
Noise in the dynamics of the networks (SDEs)
Coefficient of noise term
0.05
Noise added after the simulation (measurement error)
None
Model of noise in microarrays (used for DREAM4)
Add normal and/or log-normal noise
Add Gaussian noise with standard dev.
0.025
Add log-normal noise with standard dev.
0.075
Normalize after adding noise (as in DREAM4)
