# Supplementary material for: Evaluating methods of inferring gene regulatory networks highlights their lack of performance for single cell gene expression data
Source: BMC Bioinformatics. 2018 Jun 19;19:232. doi: 10.1186/s12859-018-2217-z (PMC6006753; doi:10.1186/s12859-018-2217-z)
Supplement: Supplementary file 2 — Figure S2. Data distributions of the single cell experimental and simulated datasets. The two experimental datasets showed evidence of zero-inflation which is a typical feature of single cell gene expression data (A & B). For the simulated datasets (C & D), zero values were added according to a probablistic scheme for drop-out events as per experimental single cell data. The simulated datasets (C & D) still have notable differences in the data distributions compared to the real single cell experiment data (A & B), despite the underlying mechanisms designed to mimic features of the single cell data. (PDF 383 kb) [file 12859_2018_2217_MOESM2_ESM.pdf]

Figure S2

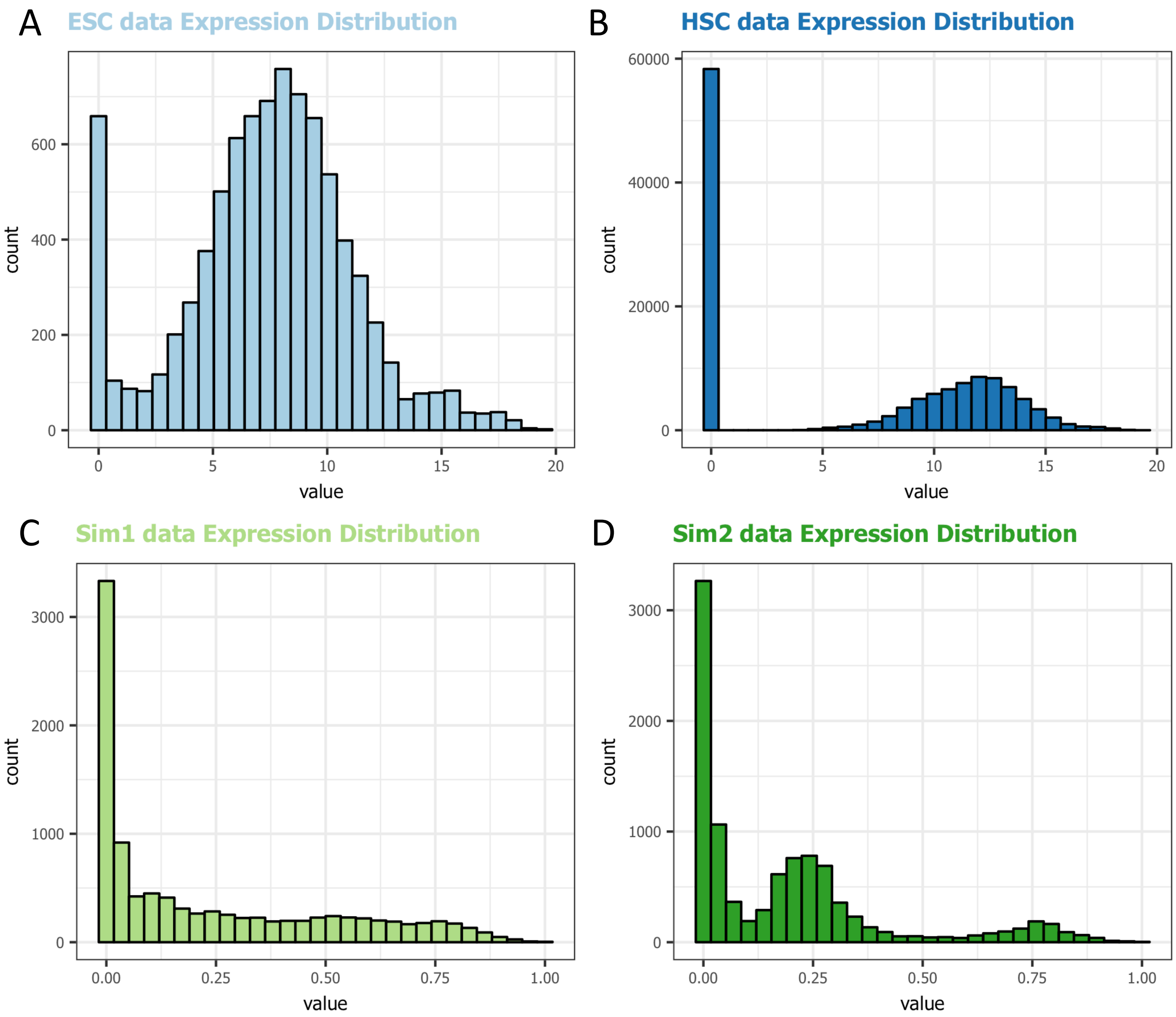
A
ESC data Expression Distribution
count
value
B
HSC data Expression Distribution
count
value
C
Sim1 data Expression Distribution
count
value
D
Sim2 data Expression Distribution
count
value
