# Supplementary material for: Evaluating methods of inferring gene regulatory networks highlights their lack of performance for single cell gene expression data
Source: BMC Bioinformatics. 2018 Jun 19;19:232. doi: 10.1186/s12859-018-2217-z (PMC6006753; doi:10.1186/s12859-018-2217-z)
Supplement: Supplementary file 3 — Table S1. Summary of metrics used to evaluate performance of each method. (DOCX 19 kb) [file 12859_2018_2217_MOESM3_ESM.docx]

**Table S1.** Summary of metrics used to evaluate performance of each method.

|  |  | Network methods | | | | | | | |
| --- | --- | --- | --- | --- | --- | --- | --- | --- | --- |
| Dataset  (# of edges in reference) | Number of Edges | Pcorr | BNs | GENIE3 | ARACNE | CLR | SCENIC | SCODE | PIDC |
| ESC (409) | Detected | 217 | 330 | 255 | 1262 | 3199 | 172 | 330 | 165 |
|  | TP | 32 | 40 | 31 | 116 | 289 | 11 | 26 | 24 |
|  | TN | 3966 | 3861 | 3927 | 3005 | 1241 | 3990 | 3847 | 4010 |
|  | FP | 185 | 290 | 224 | 1146 | 2910 | 161 | 304 | 141 |
|  | FN | 377 | 369 | 378 | 293 | 120 | 398 | 383 | 385 |
|  | Precision | 0.147 | 0.121 | 0.122 | 0.092 | 0.090 | 0.064 | 0.079 | 0.145 |
|  | Recall | 0.078 | 0.098 | 0.076 | 0.284 | 0.707 | 0.027 | 0.064 | 0.059 |
|  | F1 score | 0.102 | 0.108 | 0.094 | 0.139 | 0.160 | 0.038 | 0.071 | 0.084 |
|  | FDR | 0.045 | 0.070 | 0.054 | 0.276 | 0.701 | 0.039 | 0.073 | 0.034 |
| HSC (126) | Detected | 220 | 227 | 171 | 464 | 269 | 120 | 203 | 114 |
|  | TP | 57 | 67 | 43 | 96 | 64 | 38 | 46 | 37 |
|  | TN | 239 | 242 | 274 | 34 | 197 | 320 | 245 | 325 |
|  | FP | 163 | 160 | 128 | 368 | 205 | 82 | 157 | 77 |
|  | FN | 69 | 59 | 83 | 30 | 62 | 88 | 80 | 89 |
|  | Precision | 0.259 | 0.295 | 0.251 | 0.207 | 0.238 | 0.317 | 0.227 | 0.325 |
|  | Recall | 0.452 | 0.532 | 0.341 | 0.762 | 0.508 | 0.302 | 0.365 | 0.294 |
|  | F1 score | 0.329 | 0.380 | 0.289 | 0.326 | 0.324 | 0.309 | 0.280 | 0.309 |
|  | FDR | 0.405 | 0.398 | 0.318 | 0.915 | 0.510 | 0.204 | 0.391 | 0.192 |
| Sim1 (263) | Detected | 15 | 230 | 173 | 4687 | 2367 | NA | 210 | 115 |
|  | TP | 0 | 9 | 6 | 253 | 136 | NA | 18 | 6 |
|  | TN | 4672 | 4466 | 4520 | 253 | 2456 | NA | 4495 | 4578 |
|  | FP | 15 | 221 | 167 | 4434 | 2231 | NA | 192 | 109 |
|  | FN | 263 | 254 | 257 | 10 | 127 | NA | 245 | 257 |
|  | Precision | 0.000 | 0.039 | 0.035 | 0.054 | 0.057 | NA | 0.086 | 0.052 |
|  | Recall | 0.000 | 0.034 | 0.023 | 0.962 | 0.517 | NA | 0.068 | 0.023 |
|  | F1 score | NaN | 0.036 | 0.028 | 0.102 | 0.103 | NA | 0.076 | 0.032 |
|  | FDR | 0.003 | 0.047 | 0.036 | 0.946 | 0.476 | NA | 0.041 | 0.023 |
| Sim2 (9) | Detected | 34 | 25 | 17 | 45 | 30 | NA | 25 | 13 |
|  | TP | 5 | 3 | 2 | 9 | 3 | NA | 6 | 1 |
|  | TN | 7 | 14 | 21 | 0 | 9 | NA | 17 | 24 |
|  | FP | 29 | 22 | 15 | 36 | 27 | NA | 19 | 12 |
|  | FN | 4 | 6 | 7 | 0 | 6 | NA | 3 | 8 |
|  | Precision | 0.147 | 0.120 | 0.118 | 0.200 | 0.100 | NA | 0.240 | 0.077 |
|  | Recall | 0.556 | 0.333 | 0.222 | 1.000 | 0.333 | NA | 0.667 | 0.111 |
|  | F1 score | 0.233 | 0.176 | 0.154 | 0.333 | 0.154 | NA | 0.353 | 0.091 |
|  | FDR | 0.806 | 0.611 | 0.417 | 1.000 | 0.750 | NA | 0.528 | 0.333 |
