# Supplementary figures and images for: Evaluating methods of inferring gene regulatory networks highlights their lack of performance for single cell gene expression data
Source: BMC Bioinformatics. 2018 Jun 19;19:232. doi: 10.1186/s12859-018-2217-z (PMC6006753; doi:10.1186/s12859-018-2217-z)

Figure S3

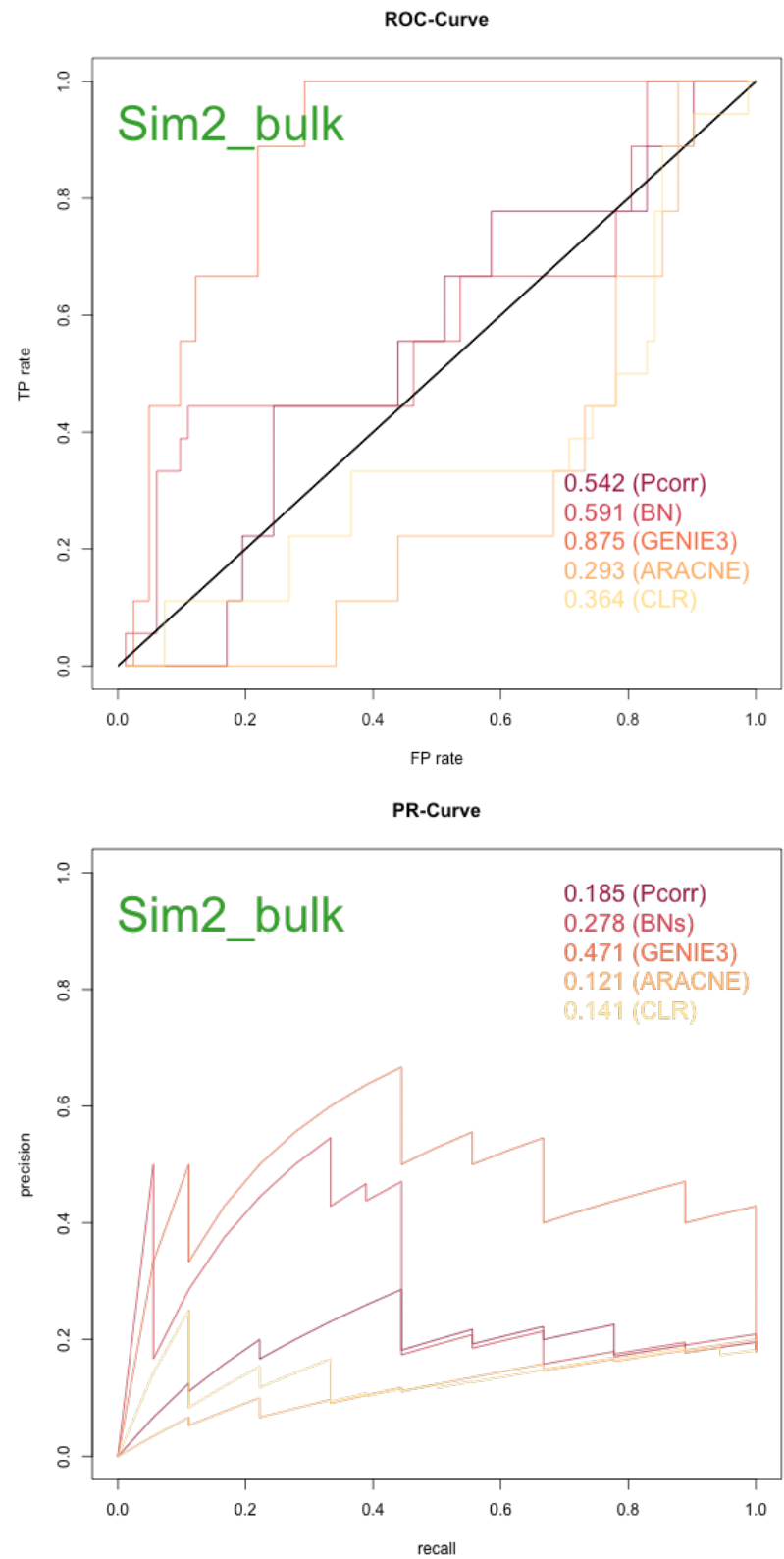

Supplement: Supplementary file 4 — Figure S3. ROC (top) and PR (bottom) curves for general methods applied to the Sim2 datasets without the drop-out effect. To examine whether poor performance observed was due to the methods, or the complexity of the datasets, we used the Sim2_bulk dataset (Sim2 without inducing drop-out). From both AUROC and AUPR scores, all five methods had improved performance on the Sim2_bulk dataset compared to the performance on Sim2 data where drop-out was included. (PDF 180 kb) [file 12859_2018_2217_MOESM4_ESM.pdf]

Figure S4

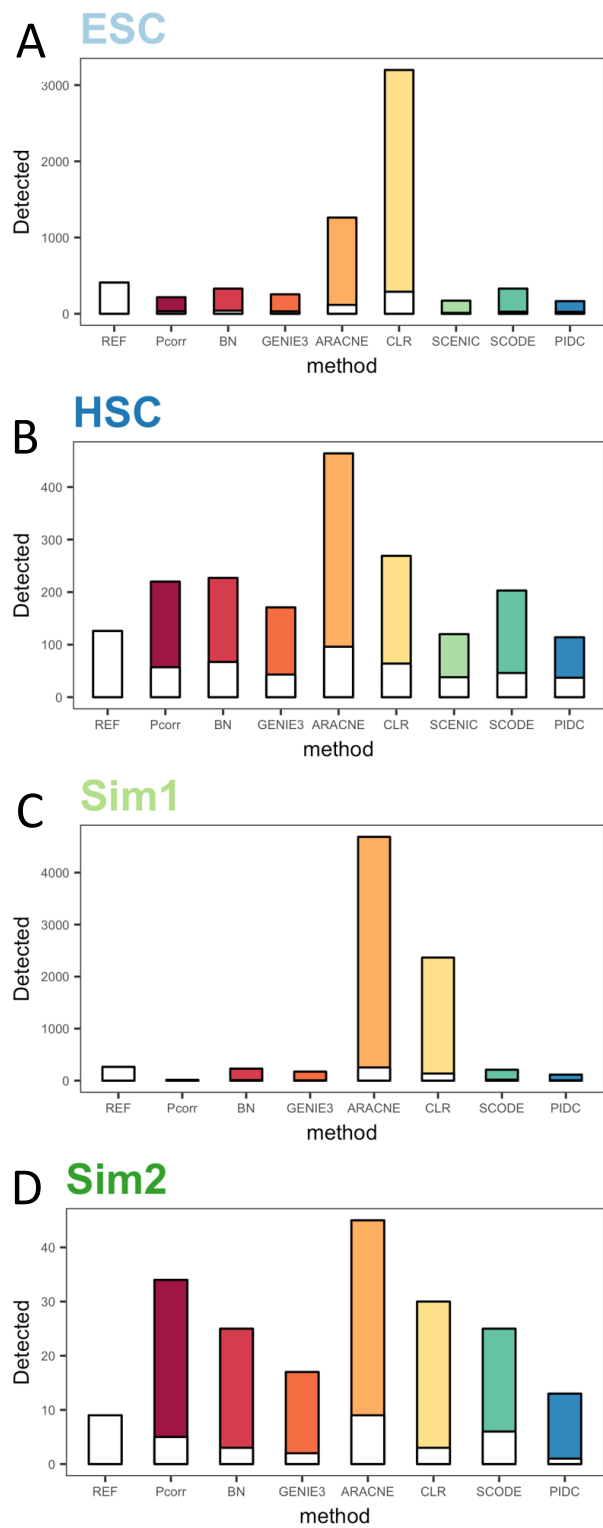

Supplement: Supplementary file 5 — Figure S4. Variable numbers of edges (and true positives) were detected for each method from the four datasets. Each bar represents the total number of edges detected (colored bar) and the number of True Positives (TPs) among them (white bar) when using the default setting of each method to apply for each dataset. The total number of edges detected varies widely for each method, and ARACNE and CLR detected far more edges than the reference networks and other methods. For the Sim2 dataset, ARACNE recovered a fully-connected network. (PDF 272 kb) [file 12859_2018_2217_MOESM5_ESM.pdf]

Figure S5

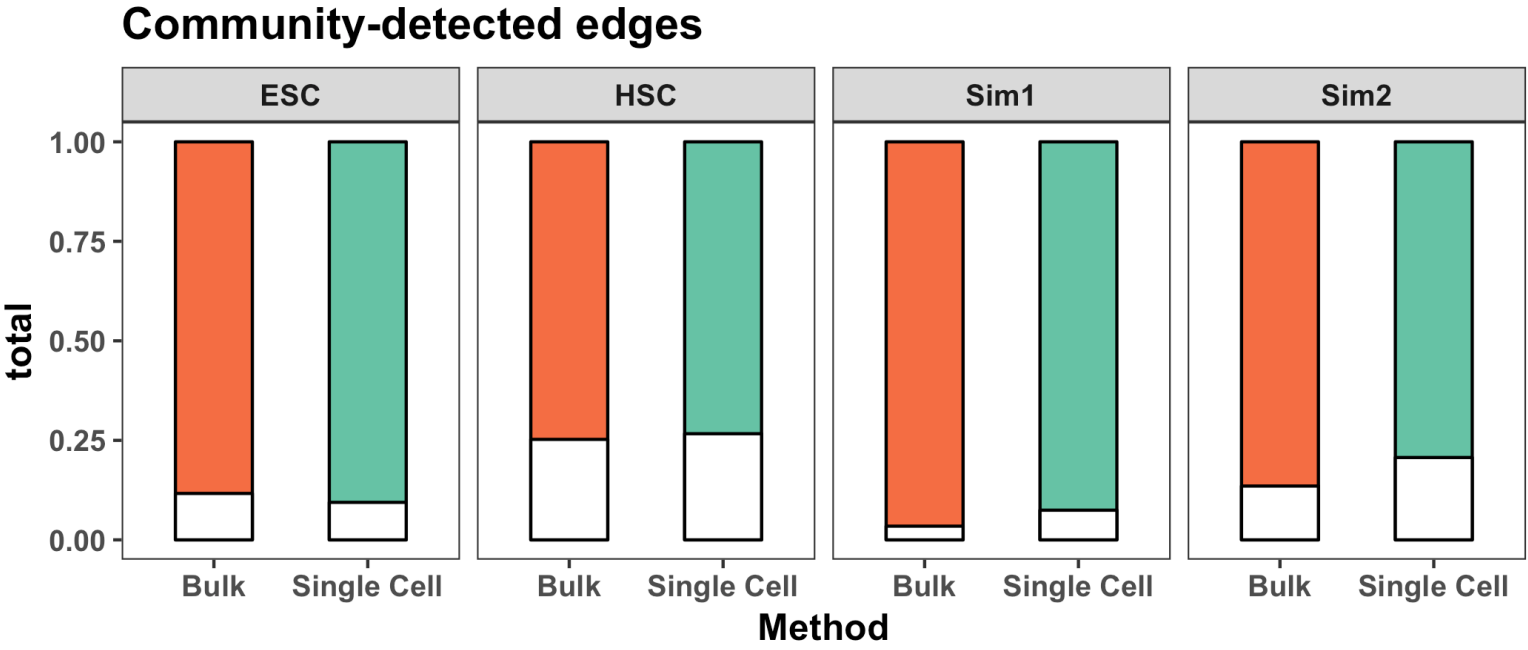

Supplement: Supplementary file 6 — Figure S5. Comparison of community-detected network by bulk sample network methods versus single cell network methods. For general methods (or ‘bulk’ methods), we obtained the union of edges from Pcorr, BN and GENIE3, and compared how this union network overlaps with the reference network. For single cell methods, we applied the same comparison by obtaining the union of SCENIC (included only for the ESC and HSC data), SCODE and PIDC. For three out of the four datasets, the union of the single cell methods had a higher recovery rate than the union of general methods. (PDF 194 kb) [file 12859_2018_2217_MOESM6_ESM.pdf]

Figure S6

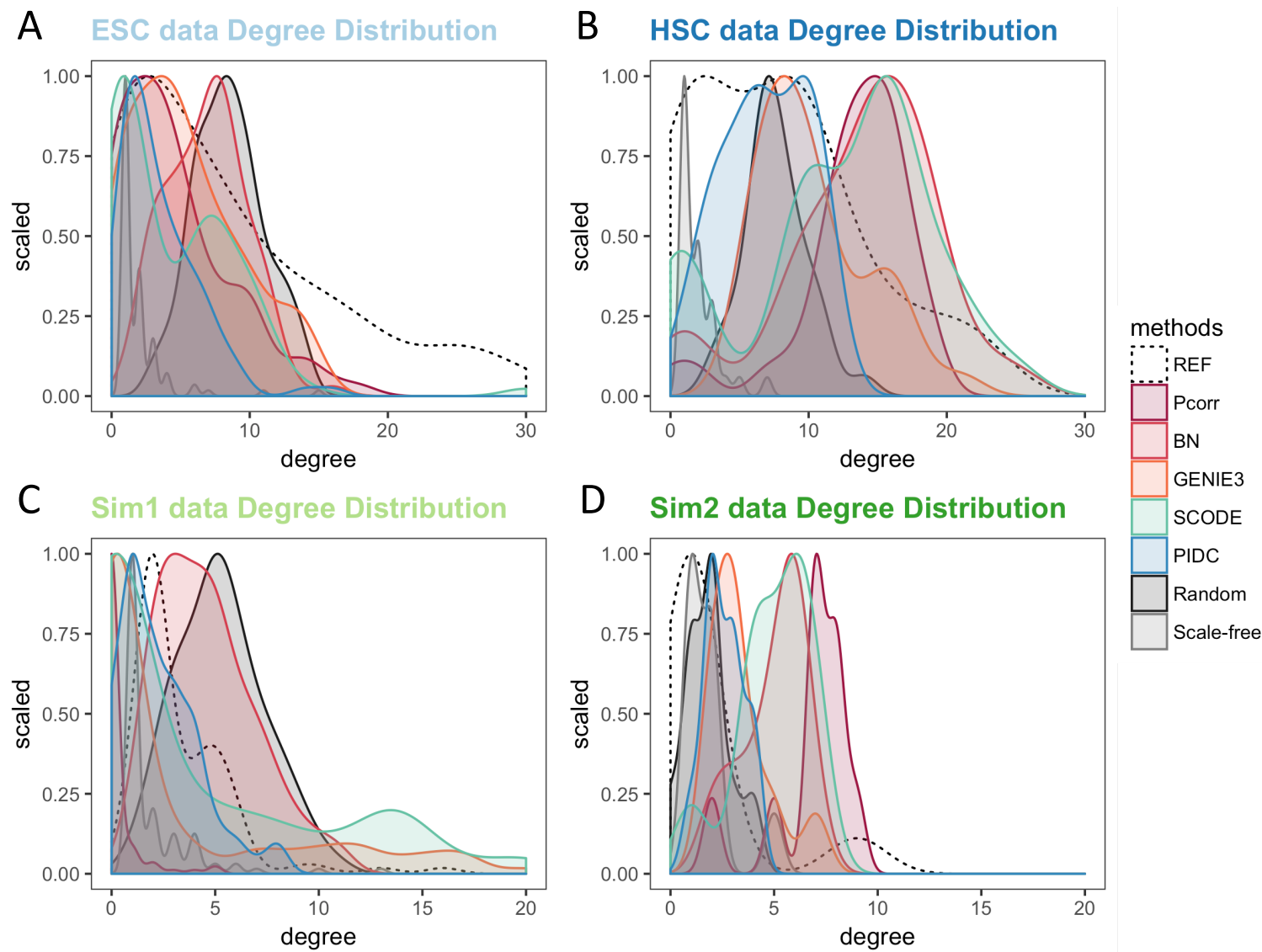

Supplement: Supplementary file 7 — Figure S6. Degree distributions of the learned networks (from Pcorr, BN, GENIE3, SCODE and PIDC), reference networks, and theoretical distributions of random graph and scale-free network. The network models were learned using default settings, and degree distributions were represented as density plots. Theoretical models of random graphs and scale-free network were generated for each dataset. For the simulated datasets, reference networks overlapped relatively well with networks with the theorectical scale-free network (C & D), while for single cell datasets, the reference networks had much wider distributions. (PDF 745 kb) [file 12859_2018_2217_MOESM7_ESM.pdf]
